# Long noncoding RNA ERLR mediates epithelial-mesenchymal transition of retinal pigment epithelial cells and promotes experimental proliferative vitreoretinopathy

**DOI:** 10.1038/s41418-021-00756-5

**Published:** 2021-03-04

**Authors:** Shuai Yang, Hui Li, Haipei Yao, Yao Zhang, Huiqian Bao, Liangjing Wu, Conghui Zhang, Min Li, Le Feng, Jingfa Zhang, Zhi Zheng, Guotong Xu, Fang Wang

**Affiliations:** 1 Department of Ophthalmology, Shanghai Tenth People’s Hospital, Tongji University, School of Medicine, Shanghai, China; 2 Tongji Eye Institute, Tongji University School of Medicine, Shanghai, China; 3 Department of Preventative Ophthalmology, Shanghai Eye Disease Prevention and Treatment Center/Shanghai Eye Hospital, Shanghai, China; 4 Nanchang University, School of Medicine, Nanchang, Jiangxi China; 5 Department of Ophthalmology, Shanghai General Hospital, Shanghai Jiao Tong University School of Medicine, Shanghai, China

**Keywords:** Cadherins, Neurological disorders

## Abstract

Proliferative vitreoretinopathy (PVR) is a disease that causes severe blindness and is characterized by the formation of contractile fibrotic subretinal or epiretinal membranes. The epithelial-mesenchymal transition (EMT) of retinal pigment epithelial (RPE) cells is a hallmark of PVR. This work aims to examine the role of a long noncoding RNA (lncRNA) named EMT-related lncRNA in RPE (ERLR, LINC01705-201 (ENST00000438158.1)) in PVR and to explore the underlying mechanisms. In this study, we found that ERLR is upregulated in RPE cells stimulated with transforming growth factor (TGF)-β1 as detected by lncRNA microarray and RT-PCR. Further studies characterized full-length ERLR and confirmed that it is mainly expressed in the cytoplasm. In vitro, silencing ERLR in RPE cells attenuated TGF-β1-induced EMT, whereas overexpressing ERLR directly triggered EMT in RPE cells. In vivo, inhibiting ERLR in RPE cells reduced the ability of cells to induce experimental PVR. Mechanistically, chromatin immunoprecipitation (ChIP) assays indicated that the transcription factor TCF4 directly binds to the promoter region of ERLR and promotes its transcription. ERLR mediates EMT by directly binding to MYH9 protein and increasing its stability. TCF4 and MYH9 also mediate TGF-β1-induced EMT in RPE cells. Furthermore, ERLR is also significantly increased in RPE cells incubated with vitreous PVR samples. In clinical samples of PVR membranes, ERLR was detected through fluorescent in situ hybridization (FISH) and colocalized with the RPE marker pancytokeratin (pan-CK). These results indicated that lncRNA ERLR is involved in TGF-β1-induced EMT of human RPE cells and that it is involved in PVR. This finding provides new insights into the mechanism and treatment of PVR.

## Introduction

Proliferative vitreoretinopathy (PVR), which occurs as a complication of rhegmatogenous retinal detachment (RRD), is a disease that causes severe blindness and is characterized by the formation of subretinal or epiretinal membranes [1, 2]. Currently, the epithelial–mesenchymal transition (EMT) of retinal pigment epithelium (RPE) is considered a hallmark of PVR pathogenesis [3]. Previous studies indicated that various growth factors/cytokines (TGF-β [4], PDGF [5, 6], EGF [7], etc.), intracellular signaling pathways (Smads [8], Wnt [7, 9], Akt2 [10], TAK1 [11], etc.), transcription factors (Snail families [4] and ZEB family [7, 9]), and microRNAs (miR29b [12] and miR204/211) play important roles in EMT in RPE cells. Antagonizing these molecules modulates the progression of PVR experimental models and thus might provide potential clinical insights [3]. However, the EMT of RPE cells is a complicated process. Our understanding of the detailed mechanisms is far from clear; thus, we still have no good solution for clinical PVR. One of the reasons might be that we did not pay much attention to another type of molecule, namely lncRNA, which constitutes the vast majority of the genome and was initially considered the “dark matter of the genome”.

LncRNAs are a class of nonprotein-coding RNAs that are longer than 200 nucleotides. Recent work has suggested that a large number of lncRNAs function as epigenetic modulators by interacting with other molecules, including mRNAs, miRNAs, or proteins. LncRNAs may act as signals, guides or scaffolds to chromatin to regulate the expression of target genes [13]. Their completely different functional mechanisms might open a new window on the understanding of disease development, including PVR. In fact, studies have indicated that lncRNAs regulate several physiological or pathological processes. The relevant function of lncRNAs in triggering EMT has also been observed in tumor metastasis and fibrotic diseases [14–17].

Given the powerful and diverse functions of lncRNAs in EMT-related diseases, we believe that lncRNAs contribute to the pathogenesis of PVR through target genes. Thus, addressing the role and mechanisms of lncRNAs in the EMT of RPE cells might provide a connection between various types of functional molecules and deepen our understanding of the underlying mechanism of this process. We recently reported that lncRNA-metastasis-associated lung adenocarcinoma transcript-1 (MALAT-1) mediates transforming growth factor (TGF)-β1-induced EMT of ARPE-19 cells partially through activating Smad2/3 signaling [18]. In this study, we further addressed the mechanisms of lncRNA in EMT of RPE cells and provided new insight into PVR by screening the expression profile of lncRNAs in RPE cells undergoing EMT. Furthermore, one of the dysregulated lncRNAs was selected and studied in detail to evaluate its role in PVR development.

## Materials and methods

### Reagents and antibodies

A mouse antihuman E-cadherin antibody (Cat. No.: 610182) was purchased from BD Bioscience (San Jose, CA, USA). Rabbit antihuman ZO-1 (Cat. No.: 61-7300), mouse antihuman alpha-smooth muscle actin (α-SMA) (Cat. No.: MA5-11547), mouse antihuman pancytokeratin (Pan-CK) antibody (Cat. No.: MA5-13203), fluorescein isothiocyanate (FITC)-conjugated anti-mouse, and FITC-conjugated antirabbit antibodies were obtained from Invitrogen (Carlsbad, CA, USA). A mouse antihuman fibronectin (Cat. No.: F7387) antibody was obtained from Sigma-Aldrich (St. Louis, MO, USA). Rabbit antihuman β-actin (Cat. No.: ab119716), rabbit antihuman TCF4 (Cat. No.: ab217668), rabbit antihuman MYH9 (Cat. No.: ab138498), and rabbit antihuman N-cadherin (Cat. No.: ab18203) antibodies were purchased from Abcam (Cambridge, MA, USA). Rabbit antihuman Smad2/3 (Cat. No.: 3102), p-Smad2/3 (Cat. No. 8828), p38 (Cat. No.: 9212), and p-p38 (Cat. No.: 9211) antibodies were purchased from Cell Signaling Technology (Danvers, MA, USA). A mouse antihuman GAPDH (Cat. No.: 30201ES20) antibody was purchased from Yeasen (Shanghai, China). TRIzol reagent and 4′,6-diamidino-2-phenylindole (DAPI) were purchased from Invitrogen (Carlsbad, CA, USA). A SuperReal PreMix Plus (SYBR Green) reagent kit was obtained from Takara Clontech (Kyoto, Japan). Other reagents, such as salt and buffer components, were of analytical grade and were obtained from Sigma-Aldrich (St. Louis, MO, USA).

### Ethics approval and consent to participate

The current research involving clinical samples was approved by the ethics committee of Shanghai Tenth People’s Hospital, and was in compliance with the Declaration of Helsinki. All subjects provided their written approval and consent.

All of the animal experiments were conducted in compliance with the Institutional Animal Care and Use Committee guidelines and the Association for Research in Vision and Ophthalmology Statement for the Use of Animals in Ophthalmic and Vision Research.

### Cell culture and clinical samples

The human RPE cell line ARPE-19 was obtained from the Eye Institute of Tongji University.

Primary human RPE (phRPE) cells were isolated from donors eyes (obtained from the Eye Bank of Shanghai Tenth People’s Hospital), and low passage cells (passages 2–4) were used in this study. Vitreous samples and subretinal membranes were collected from patients with PVR grade D (*n* = 8) undergoing vitrectomy. Vitreous samples from donor eyes (*n* = 4) were also collected and stored at −80 °C. Information on the PVR patients and the donors is listed in Tables S1 and S2.

ARPE-19 cells and phRPE cells were cultured in DMEM/F12 culture media (Gibco, Life Technologies) with 10% fetal bovine serum (FBS) (Gibco, Life Technologies) at 37 °C in a humidified incubator containing 5% CO_2_. Culture media were changed every 2–3 days. For further experiments, the cells were trypsinized and seeded in 6- or 12-well plates and cultured. Thereafter, the cells were starved for 16 h and then stimulated with 10 ng/ml TGF-β1 (Gibco, Life Technologies) or exposed to vitreous samples for various time periods. In some experiments, cells were transfected with an siRNA by using Lipofectamine® 3000 reagent (Invitrogen, Life Technologies) according to the manufacturer’s instructions. The siRNA and negative control (NC) siRNA sequences (Table S3) were synthesized by GenePharma (Shanghai, China). In some experiments, cells were transfected with lentiviral vectors at multiplicities of infection (MOIs) of 10. Lentiviral vectors were synthesized by Genechem (Shanghai, China).

### LncRNA and mRNA microarray analysis

ARPE-19 cells were treated with 10 ng/ml recombinant TGF-β1 or a solvent control for 48 h, and then the cells were harvested. Total RNA was extracted with TRIzol reagent (Invitrogen), transcribed into fluorescent cRNA with a Quick Amp labeling kit (Agilent Technologies, Palo Alto, CA), and hybridized to a Human LncRNA Array v3.0 (8 × 60 K, ArrayStar, Rockville, MD). Microarrays were scanned with an Agilent Scanner G2505B, and microarray images were analyzed using Agilent Feature Extraction software. The experiment was performed in triplicate. The threshold values we used to define upregulation or downregulation were fold change >2 and *P* < 0.05.

### 5′ and 3′ rapid amplification of cDNA ends (RACE)

A SMARTer™ RACE cDNA amplification kit (Clontech, Palo Alto, CA) was used according to the manufacturer’s instructions to determine the transcriptional initiation and termination sites of ERLR. The nested gene-specific primers and the gene-specific primers used for the RACE analysis are listed in Table S4. RACE products were cloned with the In-Fusion HD Cloning Kit (Clontech, Palo Alto, CA), and the resultant vectors (at least 10 for both 5′ and 3′ RACE products) were sequenced. Each experiment was performed three times.

### Isolation of cytoplasmic, nuclear, and polyadenylated RNAs

Cytoplasmic and nuclear RNAs were isolated and purified using a PARIS™ kit (Invitrogen) according to the manufacturer’s instructions. RNAs were reverse transcribed into cDNA. Polymerase chain reaction (PCR) was performed using cytoplasmic and nuclear cDNA as templates. The expression levels of the target genes, ERLR, U2, and β-actin, were detected through 1.5% agarose gel electrophoresis. The primers used in the PCRs are listed in Table S5. Experiment was independently performed three times.

### Fluorescent in situ hybridization (FISH)

A specific FISH probe for ERLR (RiboBio, Guangzhou, China) was designed and produced. FISH probes for human U6 (which was only expressed in the nucleus) and 18S RNA mix (which was only expressed in the cytoplasm) were also synthesized (RiboBio, Guangzhou, China) as negative and positive controls, respectively. A FISH kit (RiboBio, Guangzhou, China) was used to conduct FISH experiments. The PVR membranes from the clinic and cultured RPE cells were fixed with 4% paraformaldehyde for 10 min at room temperature. After washed with PBS thrice and permeabilized with 0.5% Triton X-100, PVR membranes or cells were pre-hybridized with pre-hybridization buffer (contain 1% blocking solution) for 30 min at 37 °C. Thereafter, PVR membranes or cells were hybridized in hybridization buffer containing specific FISH probes (2.5 μl, 20 μM) at 37 °C overnight. Afterwards, the PVR membranes or cells were washed in gradient decreasing concentration of SSC buffer at 42 °C (4×SSC buffer for 5 min trice, 2×SSC buffer for 5 min, 1×SSC buffer for 5 min). For the PVR membranes, IF staining for Pan-CK was conducted after FISH according to the following IF protocal. After counterstaining with DAPI, we mounted and visualized the stained coverslips under a confocal microscope (Carl Zeiss, LSM710, Jena, Germany). Experiment was independently performed three times.

### Real-time quantitative PCR

Total RNA was extracted at the indicated times with a TRIzol reagent kit, and cDNA was prepared using a PrimeScript™ RT reagent kit (Takara Clontech, Kyoto, Japan). Real-time PCR was performed in triplicate with a SuperReal PreMix Plus (SYBR Green) kit (Takara Clontech, Kyoto, Japan) on a CFX Connect real-time system (BioRad, CA, USA). Each reaction contained 12.5 μl of 2×SYBR® Premix Ex Taq™ (with SYBR Green I) and 300 nM oligonucleotide primers (Table S5) that were synthesized by Generay Corp. (Shanghai, China), and 1 μl of cDNA in a final volume of 25 μl. Thermal cycling conditions included an initial denaturation step at 95 °C for 30 s and 40 cycles of 95 °C for 5 s and 60 °C for 30 s. RNA expression was normalized to GAPDH mRNA levels. Each independent experiment included 3 replicates. 3 independent experiments were conducted. The average relative expression (target gene versus GAPDH) of control group was normalized to 1. In figures presenting qRT-PCR results, each dot represents the average of 3 technical replicates from a single independent experiment.

### Western blot (WB) analysis

After treatment was administered, phRPE cells were lysed on ice for 30 min in RIPA buffer (Beyotime, Shanghai, China) supplemented with phenylmethylsulfonyl fluoride and PhoSTOP EASY pack phosphatase inhibitor (Roche, Mannheim, Germany). Lysates were clarified through centrifugation at 12,000 rpm for 5 min. Total protein concentration was quantified using a bicinchoninic acid assay kit (Pierce, Rockford, IL, USA). Proteins (50 μg) were loaded on 6% and 10% gels and separated by sodium dodecyl sulfate polyacrylamide gel electrophoresis. Proteins were subsequently transferred to nitrocellulose membranes (BioRad, CA, USA). The membranes were blocked using 5% bovine serum albumin (BSA, Sigma Aldrich, MO, USA) in phosphate-buffered saline (PBS) for 45 min at room temperature to avoid nonspecific binding. The membranes were then incubated at 4 °C overnight with primary antibodies diluted in PBS containing 2% BSA and 0.1% Tween-20 (PBS-T) (ZO-1: 1:2000; E-Ca: 1:500; FN: 1:3000; α-SMA: 1:1000; N-Ca: 1:500; MYH9:1:5000; TCF4: 1:500; β-actin: 1:5000 and GAPDH: 1:10000). Then, membranes were rinsed with PBS-T three times and incubated with IRDye® 680LT goat antirabbit or IRDye® 800CW goat anti-mouse secondary antibodies (1:5000, Li Cor Biosciences, NE, USA) at room temperature for 1 h. The membranes were washed with PBS-T three times, and the bound antibody was detected with an Odyssey infrared imaging system (Li Biosciences, NE, USA). The band intensities were analyzed with Odyssey software and were normalized to β-actin or GAPDH. Relative protein expression was calculated with the control group taken as 1. Experiment was independently performed three times

### Immunofluorescence (IF) analysis

PhRPE cells were seeded and cultured in a 24-well plate inlaid with glass coverslips. After siRNA transfection and TGF-β treatment were performed, the cells were washed and fixed in cold acetone for 5 min. Then, cells were washed with PBS three times, blocked with 2% BSA for 1 h at room temperature, and incubated with primary antibodies (ZO-1:1:100; E-Ca: 1:50; FN: 1:200;α-SMA: 1:100 and Pan-CK: 1:100) overnight at 4 °C. After rinsing with PBS three times, we further incubated the coverslips with FITC-conjugated secondary antibodies (1:2000) for 1 h at room temperature. After counterstaining with DAPI, we mounted and visualized the stained coverslips under a confocal microscope (Carl Zeiss, LSM710, Jena, Germany). The experiments were repeated three times.

### Transwell migration assay

PhRPE cells transfected with an ERLR siRNA or an NC siRNA were incubated with TGF-β1 (10 ng/ml) for 48 h. The cells were trypsinized, and 5 × 10^4^ cells were seeded in the upper chamber of 24-well Transwell plates (8 mm pore size, Costar, Conning, CA, USA) in 100 μl of DMEM/F12 containing 0.5% FBS. The lower compartment was filled with 600 μl of DMEM/F12 with 10% FBS. The chambers were then incubated at 37 °C for 18 h. After the cells on the upper surface of the filter were removed, the migrated cells on the lower surface of the culture inserts were fixed with methanol and stained with 0.1% crystal violet for 30 min. The number of migrated cells in each chamber was then determined by counting five random fields. The experiments were repeated three times.

### Gel contraction assay

First, 24-well plates were coated with 1% BSA solution and incubated for 2 h at 37 °C. PhRPE cells were trypsinized and resuspended in serum-free DMEM/F12 culture medium. Collagen I (3 mg/ml; rat tail; Gibco, Life Technologies), 6.25 × DMEM/F12, a suspension of RPE cells (5 × 10^6^ cells/ml in DMEM/F12), 1 M NaOH, and sterile water were mixed on ice with volumes of 333, 73.6, 40, 8.3, and 45.1 μl, respectively. The final concentration of type I collagen was 2 mg/ml, and the final cell density was 4 × 10^5^/ml. Thereafter, the mixture was in a final volume of 500 μl and was added to the BSA-coated well of the 24-well plate. The plate was then incubated at 37 °C with 5% CO_2_ for 1 h until the gel solidified. The collagen gel was subsequently freed from the wall of the wells with a micropipette. Fresh serum-free DMEM/F12 (500 μl) was then added to the top of the gel. Digital photos of the gels were captured after 48 h. The area of the gels was evaluated with ImageJ software (NIH, Bethesda, MD, USA), and the relative area of the gels at the indicated times was normalized to its area at 0 h. Experiment was independently performed three times.

### PVR rabbit model

The ERLR-shRNA lentivirus (lv-sh-ERLR and lv-sh-ERLR-2) and NC shRNA lentivirus (lv-sh-NC) were constructed and transfected into phRPE cells. The siRNA sequences used in the shRNA vector are listed in Table S6. The knockdown efficiency was confirmed through RT-PCR 48 h after transfection.

An open label animal study is conducted. Pigmented rabbits (about 2 kg, 3-month-old) were housed at the Animal Center of Shanghai Tenth People’s Hospital. All of the animal experiments were conducted in compliance with the Institutional Animal Care and Use Committee guidelines and the Association for Research in Vision and Ophthalmology Statement for the Use of Animals in Ophthalmic and Vision Research. In each group (randomly assigned by random number table), 6 rabbits from 2 independent experiments (3 rabbits in each independent experiment) were included. The rabbits were acclimated at room temperature for 1 week, anesthetized with intraperitoneal injection of sodium pentobarbital (30 mg/kg), and injected intravitreally with 0.2 ml of C_3_F_8_. After 5 days, the cultured phRPE cells were transfected with lv-sh-NC or lv-sh-ERLR. After 48 h (7 days after C_3_F_8_ injection), phRPE cells were trypsinized and resuspended. The rabbits were anesthetized and intravitreally injected with 0.1 ml of 2.5 × 10^6^/ml cells combined with 50 ng/ml recombinant human platelet derived growth factor-BB (PDGF-BB, R&D Systems). All of the injections were performed in the left eye.

The fundus was examined with a SuperQuad 160 fundus lens (Volk, OH, USA) after 7, 14, 21, and 28 days. PVR was graded using the Fastenberg scale: stage 0, normal fundus; stage 1, the presence of ERM; stage 2, ERM with focal retinal traction or abnormal vessel appearance; stage 3, localized retinal detachment (RD); stage 4, extensive RD of at least two quadrants without complete RD; and stage 5, complete RD. Fundus images were captured with an iPhone 7 smartphone under a microscope (Leica EZ4, Germany). An ultrasonic B scan was performed at the indicated times. After 28 days, the rabbits were euthanized by intravenous injection of sodium pentobarbital at 100 mg/kg, and their eyes were enucleated. The eyes were fixed with 4% paraformaldehyde and embedded in paraffin. Sections (10 μm) were prepared and subjected to hematoxylin and eosin (H&E) staining and immunofluorescent staining for α-SMA.

### Chromatin immunoprecipitation (ChIP) assay

ChIP was performed using a Pierce Magnetic ChIP kit (Cat. NO.: 26157, Thermo Fisher, USA) according to the manufacturers’ instructions. In brief, 4 × 10^6^ cells were used in the experiment. Crosslinked chromatin was broken into 150- to 1000-bp fragments by MNase digestion. The chromatin was then immunoprecipitated with 2 μg of rabbit anti-TCF4 antibody (Abcam ab217668) or 2 μg of rabbit IgG with rotation overnight at 4 °C. Then, 20 μl of magnetic beads were added into each tube, and the tubes were incubated at 4 °C for 2 h with mixing. The magnetic beads were washed with IP wash buffer five times and then eluted. After purification, the immunoprecipitated chromatin was analyzed by semiquantitative-PCR and qRT-PCR. For semiquantitative-PCR, the expression levels of the DNA products were detected through 1.5% agarose gel electrophoresis. Primer pairs used in PCRs were designed within the promoter region (Table S7, Fig. 5A).

### RNA pull-down

ERLR was transcribed in vitro and biotin-labeled with T7 RNA polymerase (Roche) and Biotin RNA Labeling Mix (Roche); it was further treated with RNase-free DNase I (Roche) to remove DNA. The resultant RNA was purified with an RNeasy Mini Kit (Qiagen, Valencia, CA). One milligram of whole-cell lysates from phRPE cells was incubated with 3 µg of purified biotinylated RNA for 1 h at 25 °C. The biotin-labeled complexes were isolated with streptavidin agarose beads (Invitrogen). The recruited proteins were subjected to liquid chromatography-mass spectrometry (LC-MS) or WB analysis. Primers containing T7 promoter sequences are listed in Table S8.

### Statistical analysis

The mean and standard error of the mean (SEM) of all of the parameters were calculated. Data were analyzed statistically through two-tailed Student’s *t* test or one-way ANOVA by post hoc comparison. Fastenberg grading scores were analyzed with the Mann–Whitney *U* test. Graphs were generated using GraphPad Prism software. *P* < 0.05 was accepted as statistically significant.

## Results

### LncRNA expression profiles are altered in RPE cells treated with TGF-β1

**Fig. 1:**
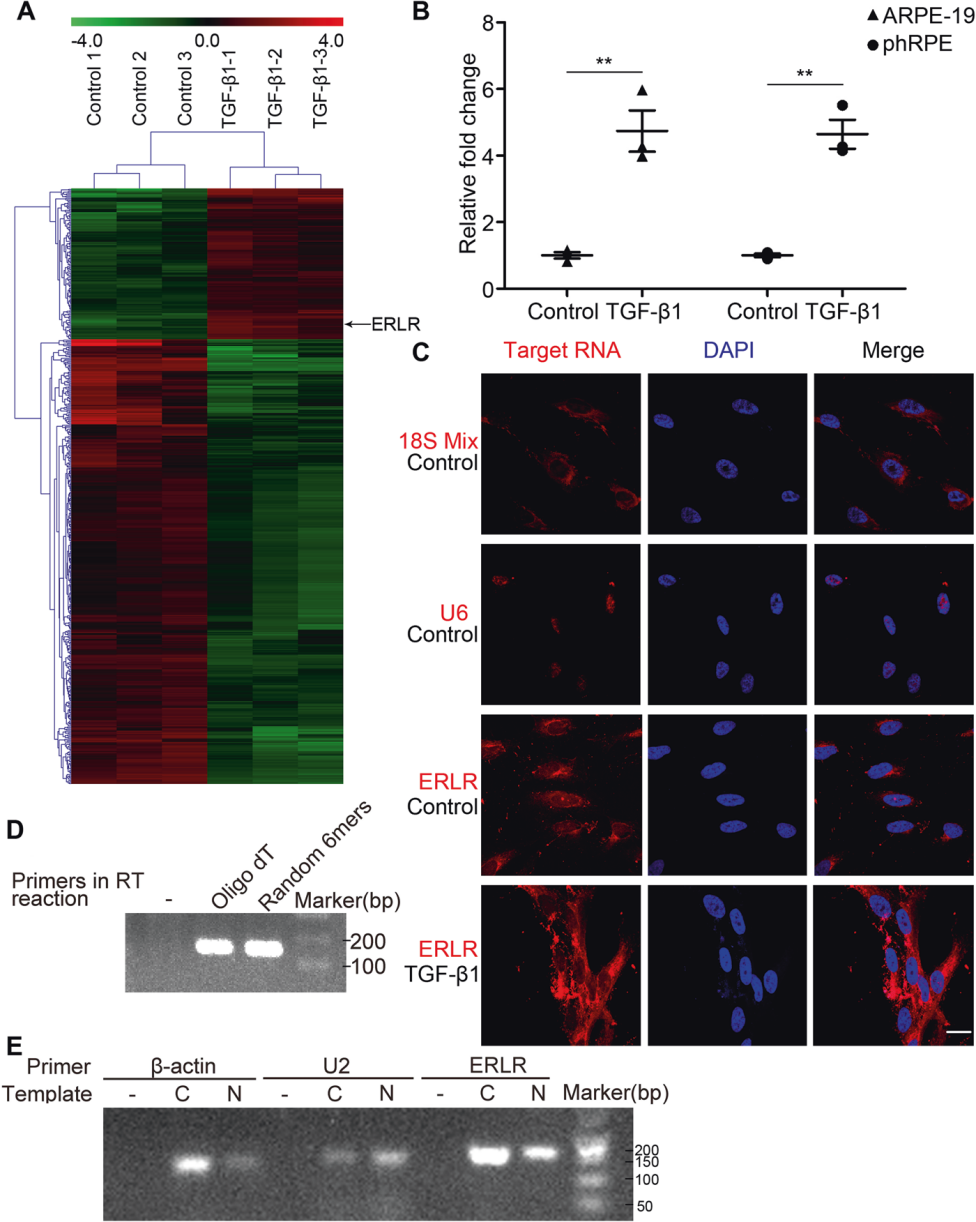
Detection and characterization of ERLR. **A** ARPE-19 cells were incubated with TGF-β1 (10 ng/ml) for 48 h. Total RNA was extracted and subjected to lncRNA microarray analysis (*N* = 3 replicates/group). Differentially expressed lncRNAs are shown. Red indicates upregulation, whereas green denotes downregulation. **B** ARPE-19 cells and phRPE cells were treated with TGF-β1 (10 ng/ml) for 48 h. ERLR expression was detected by RT-PCR, and the relative fold changes were normalized to the expression of GAPDH (Data are presented as means ± SEM. *N* = 3 independent experiments/group). **P < 0.01 by two-tailed Students’s *t* test. **C** A FISH assay was performed to observe ERLR expression in phRPE cells treated with or without TGF-β1 (10 ng/ml) for 48 h. Mixed 18S RNA (exclusively expressed in the cytoplasm), and U6(exclusively expressed in the nucleus) were included as control. One of the representative results is presented. **D** Total RNA from phRPE was reverse transcribed by different primers (no primer, oligo dT, and random 6-mers). The resulting cDNAs were examined by PCR to detect ERLR expression. Agarose gel electrophoresis was used to visualize the PCR product. One of the representative results is presented. **E** Cytoplasmic (C) and nucleic (N) RNAs were separated and reverse transcribed to generate cDNA. A PCR experiment was conducted to detect the expression of β-actin, U2, and ERLR in both cytoplasmic and nucleic cDNA. A reaction without template cDNA (−) was conducted as a negative control. Agarose gel electrophoresis was used to visualize the PCR product. One of the representative results is presented.

A lncRNA microarray was used to identify differentially expressed lncRNAs in RPE cells stimulated with TGF-β1. A total of 525 lncRNAs exhibited significant changes (fold change>2, *P* < 0.05) after TGF-β1 treatment. Of these lncRNAs, 133 were upregulated and 392 were downregulated (Fig. 1A, Supplementary File 1). The microarray data have been deposited in the GEO database with the accession number GSE105053.

We then selected the top 20 most upregulated lncRNAs according to the microarray results and confirmed their expression by RT-PCR in TGF-β1-treated phPRE cells. We found that all 20 lncRNAs were upregulated (Table S9). Of these lncRNAs, lncRNA-LINC01705-201 (ENSG00000232679.2, ENST00000438158.1) was among the top 3 upregulated lncRNAs with the highest expression abundance (Table S9) and was thus selected for further study. TGF-β1 significantly increased the expression of lncRNA- LINC01705-201 in both ARPE-19 and phRPE cells (Fig. 1B). We hypothesized that this lncRNA might play an essential role in the EMT of RPE cells. Thus, we named it EMT-related lncRNA in RPE cells (ERLR).

### Characterization of the lncRNA ERLR

To explore the cellular location of ERLR, FISH probes for ERLR, mixed 18S RNA (exclusively expressed in the cytoplasm), and U6 (exclusively expressed in the nucleus) were synthesized. FISH results indicated that ERLR is primarily expressed in the cytoplasm compared with the nucleus (Fig. 1C). Reverse transcription PCR revealed that ERLR is a polyA^+^ RNA (Fig. 1D). To further confirm its cellular location, we separated the cytoplasmic and nuclear RNAs and detected ERLR expression through PCR. β-actin and U2 genes, which are expressed in the cytoplasm and nucleus, respectively, were used as controls. The results showed that ERLR was mainly expressed in the cytoplasm (Fig. 1E). We then performed RACE and successfully obtained full-length ERLR (Fig. S1A). Heterogeneity at the 3′-end of ERLR was discovered as we obtained three RACE products with slight differences at the 3′-end (Fig. S1B–D). The longest transcript (Fig. S1B) was cloned and used in following over-expressing experiments. To confirm that ERLR was not a protein-coding transcript, three different software programs were applied to predict the coding potential of ERLR (Fig. S2A–C). The results revealed that ERLR does not have protein-coding potential. In addition, we searched the UCSC Genome Browser (Human Dec. 2013 (GRCh38/hg38) Assembly). ERLR is located on chromosome 1 (q41) and is highly conserved across different species (Fig. S2D).

### In phRPE cells, knockdown of ERLR attenuates TGF-β1-induced EMT and inhibits migration and collagen gel contraction

**Fig. 2:**
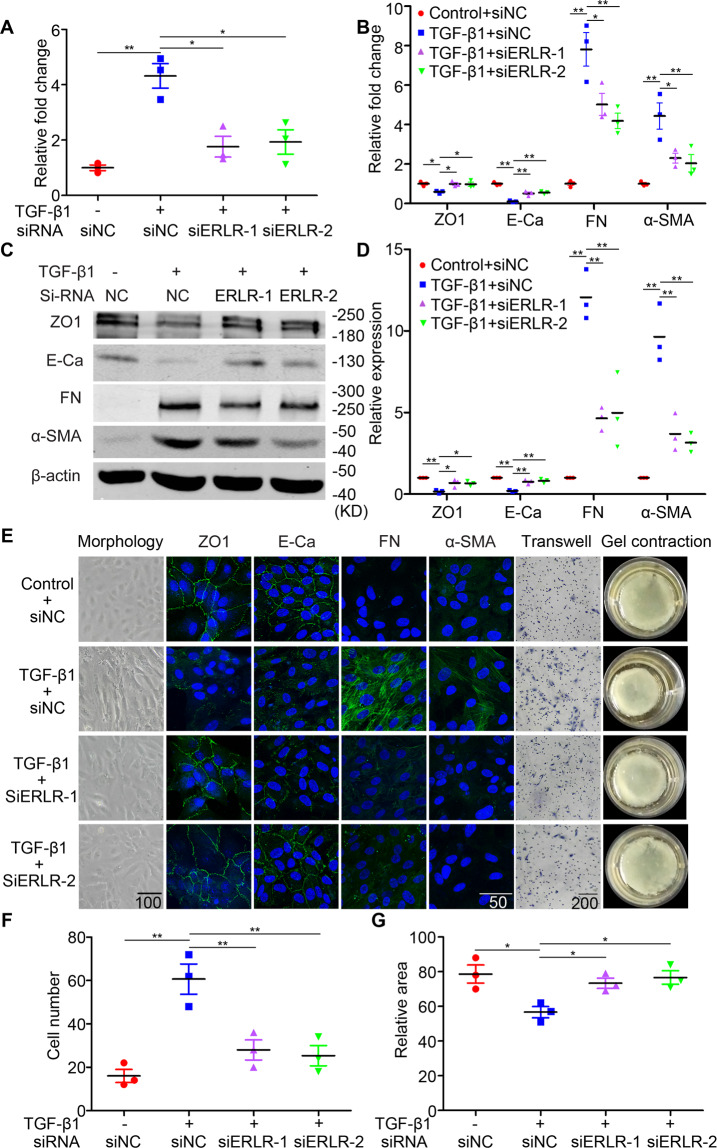
ERLR knockdown attenuates TGF-β1-induced EMT in RPE cells. PhRPE cells were transfected with two ERLR siRNAs (SiERLR-1 and SiERLR-2) or a negative control siRNA (Si-NC) and then treated with or without TGF-β1 (10 ng/ml) for 48 h. **A** Expression levels of ERLR were detected by RT-PCR, and the relative fold changes were normalized to the expression of GAPDH (*N* = 3 independent experiments/group). **P < 0.01 and *P < 0.05 by one-way ANOVA and post hoc Bonferroni’s test. **B** Expression levels of EMT-related markers (ZO-1, E-cadherin, fibronectin, and α-SMA) were detected by RT-PCR, and the relative fold changes were normalized to GAPDH (*N* = 3 independent experiments/group). **P < 0.01 and *P < 0.05 by one-way ANOVA and post hoc Bonferroni’s test. **C** EMT-related markers were detected by WB. One of the representative results is presented. **D** The band intensities in WB results were analyzed and normalized to β-actin (*N* = 3 independent experiments/group). **P < 0.01 and *P < 0.05 by one-way ANOVA and post hoc Bonferroni’s test. **E** Column 1 presents the appearances of the cells under a microscope. Columns 2–5 present the EMT-related markers detected by IF. Column 6 depicts the cells subjected to the Transwell migration assay. A representative field of vision is captured. Column 7 shows the cells subjected to the collagen gel contraction assay. After the mixture of the cells and collagen gel type I was solidified, culture medium was added. The gels were then cultured for another 48 h, and images were captured. 3–4 independent experiments were conducted. One of the representative results is presented. **F** The number of migrated cells in the Transwell migration assay was quantified by counting the average cell number in five random fields of vision (*N* = 3 independent experiments/group). **P < 0.01 by one-way ANOVA and post hoc Bonferroni’s test. **G** The area of the collagen gel was calculated by ImageJ software and was standardized to the original area (*N* = 3 independent experiments/group). *P < 0.05 by one-way ANOVA and post hoc Bonferroni’s test. All data are presented as means ± SEM.

We explored the role of ERLR in TGF-β1-induced EMT of RPE cells by knocking down ERLR expression via siRNA. phRPE cells were transfected with two ERLR-specific siRNAs (siERLR-1 and siERLR-2) or an NC siRNA (si-NC) and were then exposed to TGF-β1 (10 ng/ml) for 48 h. ERLR expression was then detected by RT-PCR. Compared with the cells transfected with si-NC, cells transfected with si-ERLRs suppressed ERLR expression by greater than 70% (Fig. 2A). RT-PCR, WB, and IF assays showed that the inhibition of ERLR significantly abrogated the TGF-β1-induced downregulation of ZO-1 and E-Ca and the upregulation of fibronectin (FN) and α-SMA (Fig. 2B–E). Furthermore, ERLR knockdown alleviated the TGF-β1-induced morphological changes in phRPE cells (Fig. 2E).

We investigated the role of ERLR in the mobility of RPE cells and the capacity of ERLR to mediate collagen gel contraction. Transwell assays revealed that ERLR knockdown significantly inhibited the migration of RPE cells (Fig. 2E, F). In the collagen gel contraction assay, TGF-β1 treatment for 48 h increased the capacity of collagen gel contraction. ERLR downregulation by siRNA in phRPE cells significantly hampered the collagen gel contraction mediated by phRPE cells (Fig. 2E, G).

### In phRPE, ERLR overexpression directly triggers EMT and promotes the migration and capacity of mediating collagen gel contraction

**Fig. 3:**
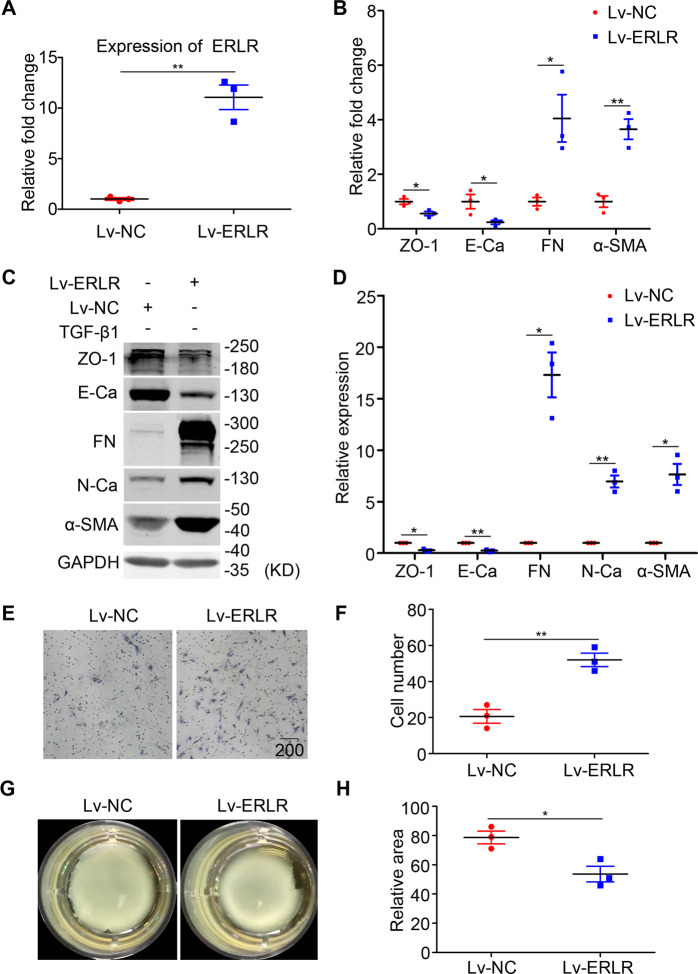
In RPE cells, ERLR overexpression triggers EMT, promotes migration, and enhances the capacity of mediating collagen gel contraction. PhRPE cells were transfected with a lentivirus overexpressing ERLR (Lv-ERLR), or a negative control lentivirus (Lv-NC). The cells were then cultured for 48 h. **A** Expression levels of ERLR were detected by RT-PCR, and the relative fold changes were normalized to the expression of GAPDH (*N* = 3 independent experiments/group). **P < 0.01 by two-tailed Students’s *t* test. **B** Expression levels of EMT-related markers (ZO-1, E-cadherin, Fibronectin, and α-SMA) were detected by RT-PCR and were normalized to GAPDH (*N* = 3 independent experiments /group). **P < 0.01 and *P < 0.05 by two-tailed Students’s *t* test. **C** Expression levels of EMT-related markers (ZO-1, E-cadherin, Fibronectin, N-cadherin and α-SMA) were detected by western blot. One of the representative results is shown. **D** The band intensities in WB results were analyzed and normalized to internal standard (*N* = 3 independent experiments/group). *P < 0.01 and *P < 0.05 by two-tailed Students’s *t* test. **E** Cells were then subjected to Transwell migration assays. A representative field of the vision is captured. **F** The number of migrated cells in the Transwell migration assay was quantified by counting the average cell number in five random fields of vision (*N* = 3 independent experiments/group). **P < 0.01 by two-tailed Students’s *t* test. **G** Cells were subjected to the collagen gel contraction assay. After the mixture of the cells and collagen gel type I was solidified, culture medium was added. The gels were then cultured for another 48 h, and their pictures were captured. **H** The area of the collagen gel was calculated using ImageJ software and was standardized to the original area (*N* = 3 independent experiments /group). *P < 0.05 by two-tailed Students’s *t* test. All data are presented as means ± SEM.

We next aimed to observe the effect of overexpressing ERLR in phRPE cells. Full-length ERLR was synthesized and inserted into a lentivirus vector (Lv-ERLR). RT-PCR results showed that ERLR expression in phRPE cells increased by ~10-fold 48 h after transfection with Lv-ERLR compared with control lentivirus (Lv-NC) (Fig. 3A). EMT markers were then detected through RT-PCR and western blot. ERLR overexpressing significantly suppressed the expression of ZO-1 and E-Ca and promoted the expression of α-SMA, FN, and N-Ca (Fig. 3B–D).

Furthermore, Transwell assays demonstrated that ERLR overexpression significantly increased the number of migrated cells (Fig. 3E, F). In addition, compared with cells infected with Lv-NC, phRPE cells overexpressing ERLR exhibited a stronger ability to mediate collagen gel contraction (Fig. 3G, H).

### Inhibition of ERLR hampers the progression of experimental PVR

We used an experimental rabbit PVR model to confirm the effect of ERLR in vivo. We constructed a shRNA lentivirus that targeted ERLR (lv-sh-ERLR), and we confirmed the knockdown efficiency in phRPE cells through RT-PCR (Fig. S3).

Pigmented rabbits received an intravitreal injection of 0.2 ml C3F8. Seven days later, 0.1 ml 2.5 × 10^6^/ml phRPE cells that had been transfected with lv-sh-ERLR or lv-sh-NC, combined with 50 ng/ml PDGF-BB were injected into the vitreous of the rabbits.

**Fig. 4:**
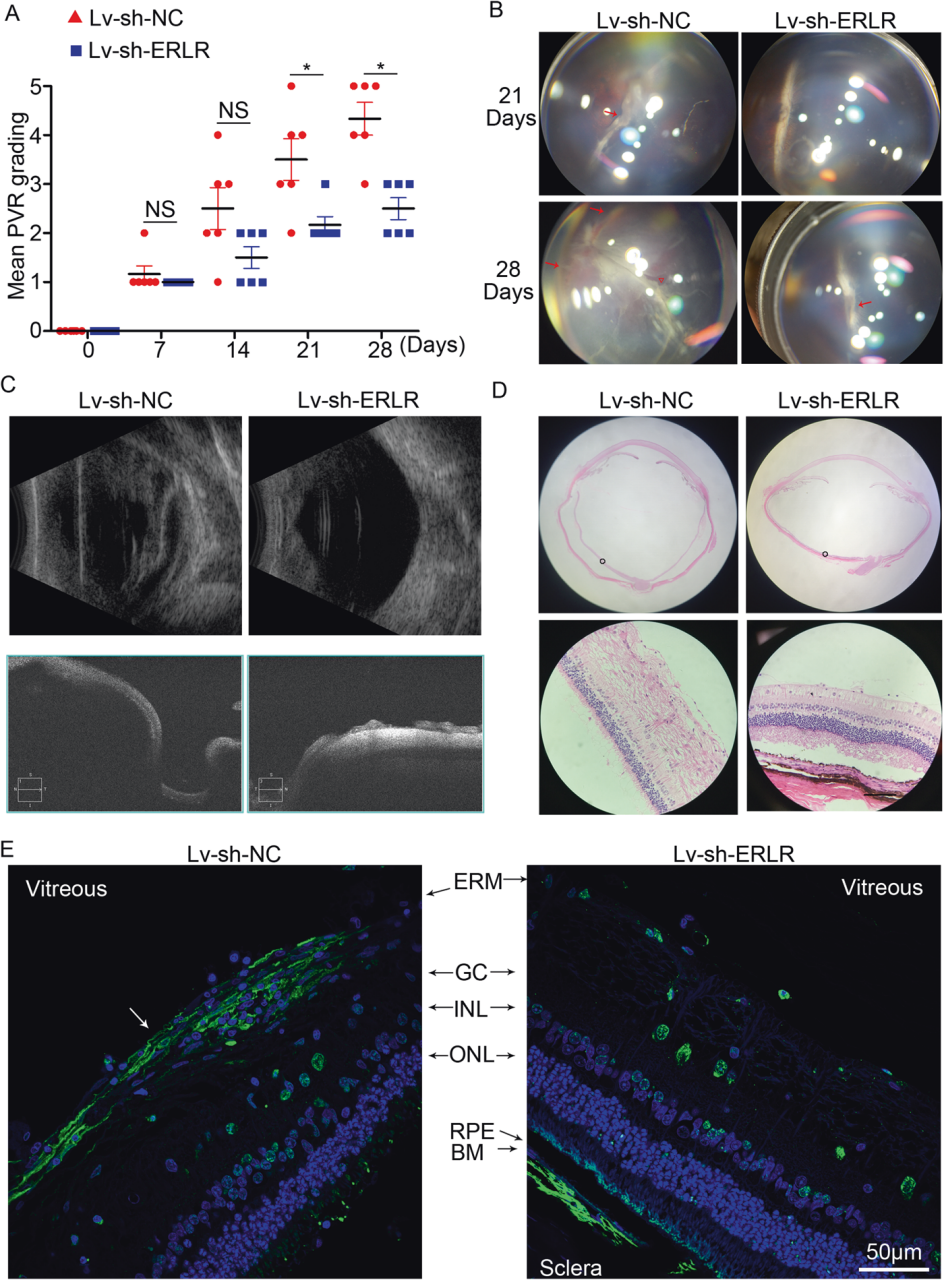
ERLR promotes PVR progression in an experimental PVR model. PhRPE cells were transfected with lv-sh-NC or lv-sh-ERLR and then were injected into the vitreous body of pigmented rabbits to induce PVR. **A** PVR severity in each group was graded according to Fastenberg’s score at the indicated times. Data are presented as means ± SEM; *N* = 6 rabbits. NS not significant, *P < 0.05 by Mann–Whitney *U* test. **B** Fundus photographs in each group were captured at the indicated times by a smartphone under a microscope with the help of a Volk SuperQuad 160 fundus lens. Localized detachment of medullary ray is shown in eyes of Lv-sh-NC group at day 21 (red arrow). Total RD with retinal folds and holes (red triangle) is shown at day 28. Focal traction and detachment (red arrows) is shown in the eyes of Lv-sh-ERLR group at day 28. **C** Ultrasound B scanning and optical coherence tomography (OCT) scanning were performed at day 28. **D** H&E staining of the rabbit eye in each group was performed and analyzed. A representative image is shown. Bar = 50 μm. **E** α-SMA expression around the retina of the rabbits was detected by immunofluorescence. The white arrow marks the α-SMA-positive epiretinal membrane. Representative results are shown.

In the following 4 weeks, PVR severity was evaluated on the basis of clinical findings that were assessed according to Fastenberg’s score. The ability to induce PVR by ERLR-inhibited phRPE cells was reduced compared with that of phRPE cells transfected with lv-sh-NC (Fig. 4A, B). Ultrasound B scanning, optical coherence tomography (OCT) scanning, and HE staining indicated that ERLR-inhibited phRPE cells failed to induce a total retinal detachment (Fig. 4C, D). Furthermore, ERLR inhibition hampered the formation of the epiretinal membrane and suppressed α-SMA expression (Fig. 4E). A similar result was achieved by another lentivirus targeting ERLR (lv-sh-ERLR-2, Figs. S3 and S4)

### The transcription factor TCF4 binds directly to the promoter region of ERLR and promotes its expression

**Fig. 5:**
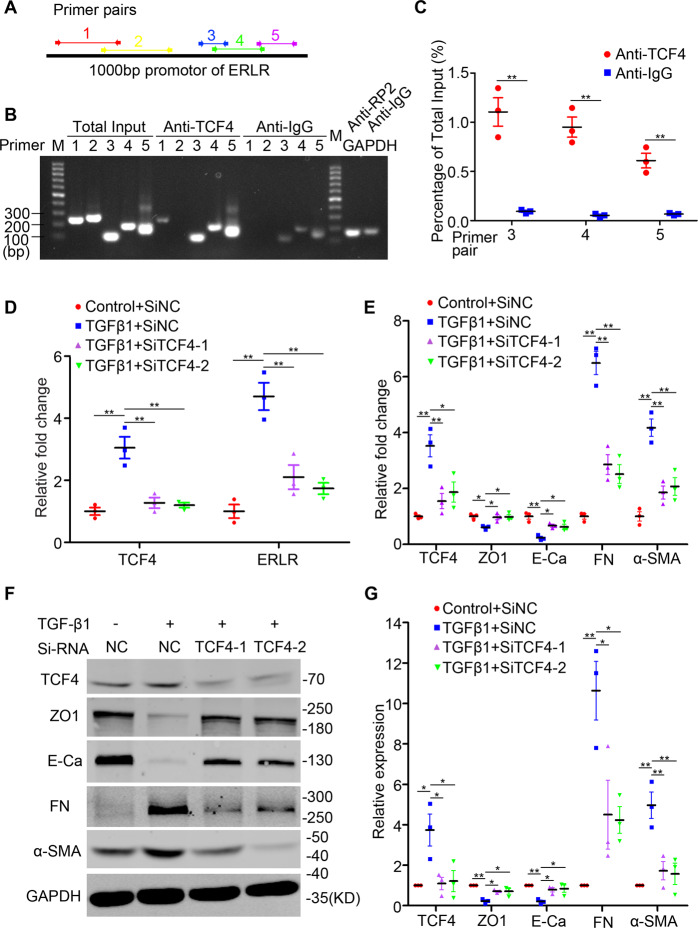
The transcription factor TCF4 directly binds to the promoter region of ERLR and promotes its expression. **A** The line represents the promoter region (1000 bp upstream) of ERLR. The regions of 5 different pairs of primers located at different regions of the promoter are depicted and were used in the following ChIP assay. **B**, **C** The chromatin of phRPE cells was immunoprecipitated with an anti-TCF4 antibody or with rabbit IgG. The resultant chromatin and total input acted as templates for semiquantitative PCR (**B**) or quantitative RT-PCR (**C**) to determine the interaction of TCF4 and the promoter region of ERLR. **B** Chromatin immunoprecipitated with anti-RP2 or rabbit IgG, acting as templates in the reaction with the primer of the GAPDH promoter, was used as a positive and negative control, respectively. **C** The relative expression of PCR products by chromatin was normalized to total input (*N* = 3 independent experiments/group). **P < 0.01 by two-tailed Students’s *t* test. **D–G** PhRPE cells were transfected with two TCF4 siRNAs (SiTCF4-1 and SiTCF4-2) or a negative control siRNA (Si-NC) and then treated with or without TGF-β1 (10 ng/ml) for 48 h. **D** TCF4 and ERLR expression was detected by RT-PCR and was normalized to the expression of GAPDH (*N* = 3/group). **P < 0.01 by one-way ANOVA and post hoc Bonferroni’s test. **E** The expression of TCF4 and EMT-related markers was detected by RT-PCR and was normalized to that of GAPDH (*N* = 3 independent experiments /group). *P < 0.05 and **P < 0.01 by one-way ANOVA and post hoc Bonferroni’s test. **F** The expression of TCF4 and EMT-related markers was detected by WB. One of the representative results is shown. **G** The band intensities in WB results were analyzed and normalized to internal standard (*N* = 3 independent experiments /group). *P < 0.05 and **P < 0.01 by one-way ANOVA and post hoc Bonferroni’s test. All data are presented as means ± SEM.

We further explored the regulatory mechanisms of ERLR. Transcription factors that potentially bind to the promoter region of ERLR were predicted by PROMO (http://alggen.lsi.upc.es/cgi-bin/promo_v3/promo/promoinit.cgi?dirDB=TF_8.3) (Fig. S5A). Of all these transcription factors, three (TCF4, Fos, and IRF1) exhibited a significant expression difference in RPE cells after treatment with TGF-β1, as shown by our microarray results (Fig. S5B). Of these three transcription factors, TCF4, a WNT signaling component capable of mediating TGF-β signaling, exhibited a significant expression correlation with ERLR based on the intensity reads from the microarray data (Fig. S5C). CNC analysis also revealed a relationship between ERLR and TCF4 (Fig. S6). We further confirmed the expression of TCF4 and ERLR in RPE cells treated with or without TGF-β1 by qPCR and compared the expression relationship of these two genes by linear regression. Similarly, a significant correlation was observed between these two genes (Fig. S5D). ChIP assays were then performed to determine whether TCF4 directly binds to the promoter region of ERLR. We designed five primer pairs in different regions within the 1000 bp upstream of ERLR (Fig. 5A, Fig. S5E, Table S7). Semiquantitative PCR and qRT-PCR were conducted with the ChIP product and five primer pairs. The results indicated that TCF4 directly bound to the promoter region of ERLR in the range of primer pairs 3–5 (approximately −420~ −50 bp upstream of ERLR) (Fig. 5B, C). To further determine the relationship of TCF4 and ERLR, two TCF4-specific siRNAs (Si-TCF4-1 and Si-TCF4-2) were synthesized and transfected into phRPE cells treated with TGF-β1. Both Si-TCF4-1 and Si-TCF4-2 significantly inhibited the TCF4 expression induced by TGF-β1. Knockdown of TCF4 inhibited TGF-β1-induced upregulation of ERLR (Fig. 5D).

We further questioned whether inhibition of TCF4 directly abrogates TGF-β1-induced EMT in phRPE cells. Two TCF4 siRNAs were transfected into phRPE cells treated with TGF-β1. RT-PCR and WB results indicated that knockdown of TCF4 significantly inhibited the TGF-β1-induced downregulation of ZO-1 and E-Ca and the upregulation of FN and α-SMA (Fig. 5E–G).

### ERLR triggers EMT by directly binding to MYH9 protein

**Fig. 6:**
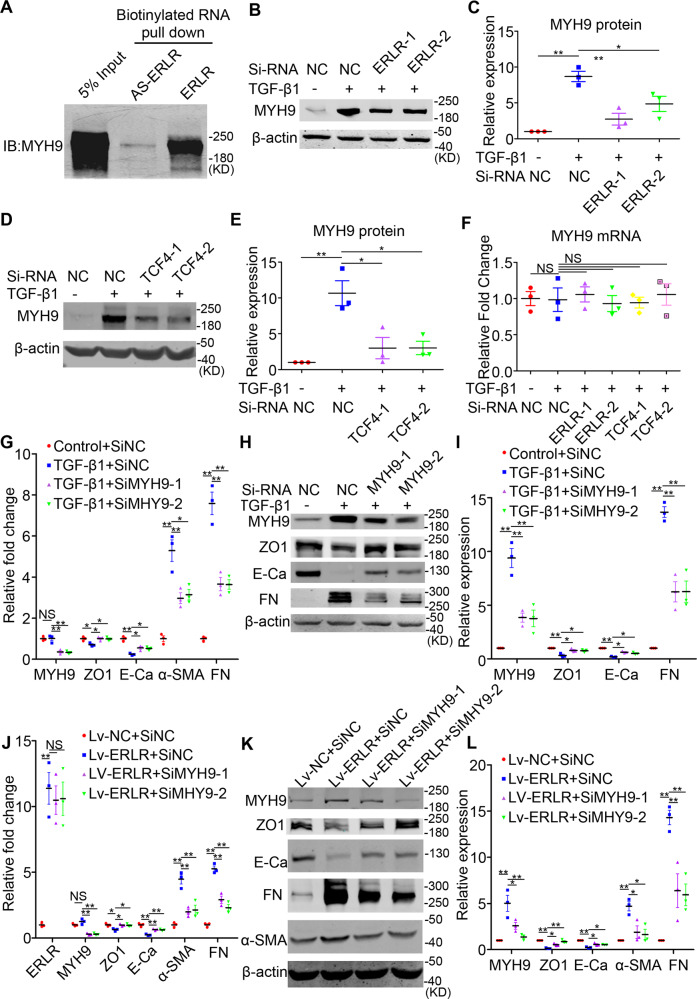
ERLR triggers EMT by directly binding to the MYH9 protein. **A** An RNA pulldown assay was performed using phRPE cells and biotinylated ERLR or antisense-ERLR. Proteins coprecipitated with biotin-labeled ERLR or antisense-ERLRs were detected by WB. One of the representative results is shown (*N* = 3 independent experiments /group). **B–F** PhRPE cells were transfected with two ERLR siRNAs (SiERLR-1 and SiERLR-2), two TCF4 siRNAs (siTCF4-1 and siTCF4-2) or a negative control siRNA (Si-NC) and then treated with or without TGF-β1 (10 ng/ml) for 48 h. **B**, **D** The expression of MYH9 protein was detected by WB. One of the representative results is shown. **C**, **E** The band intensities in the WB results were analyzed and normalized to β-actin (*N* = 3 independent experiments /group) in **B**, **D**, respectively. **P < 0.01 and *P < 0.05 by one-way ANOVA and post hoc Bonferroni’s test. **F** The expression of MYH9 mRNA was detected by qRT-PCR and was normalized to that of GAPDH (*N* = 3 independent experiments /group). No significant difference by one-way ANOVA and post hoc Bonferroni’s test. **G**–**I**: PhRPE cells were transfected with two MYH9 siRNAs (SiMYH9-1 and SiMYH9-2) or a negative control siRNA (Si-NC) and then were treated with or without TGF-β1 (10 ng/ml) for 48 h. **G** Expression levels of MYH9 and EMT-related markers were detected by RT-PCR, and the relative fold changes were normalized to GAPDH (*N* = 3 independent experiments /group). **P < 0.01 and *P < 0.05 by one-way ANOVA and post hoc Bonferroni’s test. **H** EMT-related markers were detected by WB. One of the representative results is shown. **I** The band intensities in WB results were analyzed and normalized to the intensity of β-actin (*N* = 3 independent experiments/group). **P < 0.01 and *P < 0.05 by one-way ANOVA and post hoc Bonferroni’s test. **J**–**L** PhRPE cells were transfected with ERLR-overexpressing lentivirus (lv-ERLR) or control lentivirus (lv-NC) and were then transfected with two MYH9 siRNAs (SiMYH9-1 and SiMYH9-2) or a negative control siRNA (Si-NC) and cultured for 48 h. J. Expression levels of MYH9 and EMT-related markers were detected by RT-PCR, and the relative fold changes were normalized to GAPDH (*N* = 3 independent experiments/group). **P < 0.01 and *P < 0.05 by one-way ANOVA and post hoc Bonferroni’s test. **K** EMT-related markers were detected by WB. One of the representative results is shown. **L** The band intensities in WB results were analyzed and normalized to the intensity of β-actin (*N* = 3 independent experiments /group). **P < 0.01 and *P < 0.05 by one-way ANOVA and post hoc Bonferroni’s test. All data are presented as means ± SEM.

To decipher the mechanisms by which ERLR triggers EMT, we first analyzed whether the phosphorylation of Smad2/3 and P38, two well-known TGF-β signaling pathways, was affected by ERLR. Western blot results showed that ERLR knockdown did not appear to affect the phosphorylation of either Smad2/3 or P38 (Fig. S7A, B). Thereafter, we investigated the possibility that ERLR regulated protein stability by direct binding. We first performed an RNA pulldown experiment combined with mass spectrometry (MS) analysis (Supplementary file 2). MYH9, a cytoskeleton-related protein capable of triggering EMT in various cancers, is one of the most abundant proteins that binds to ERLR. WB for the RNA pulldown results confirmed the binding of ERLR with MYH9 (Fig. 6A). We further confirmed the regulatory relationship between ERLR or TCF4 and MYH9. Knocking down ERLR or TCF-4 led to decreased expression of MYH9 at the protein level but not at the mRNA level (Fig. 6B–F). Moreover, knocking down MYH9 alleviated TGF-β1-induced EMT in phRPE cells (Fig. 6G–I). Furthermore, knocking down MYH9 also alleviated ERLR-overexpression-induced EMT in phRPE cells (Fig. 6J–L).

### ERLR expression is associated with clinical PVR

**Fig. 7:**
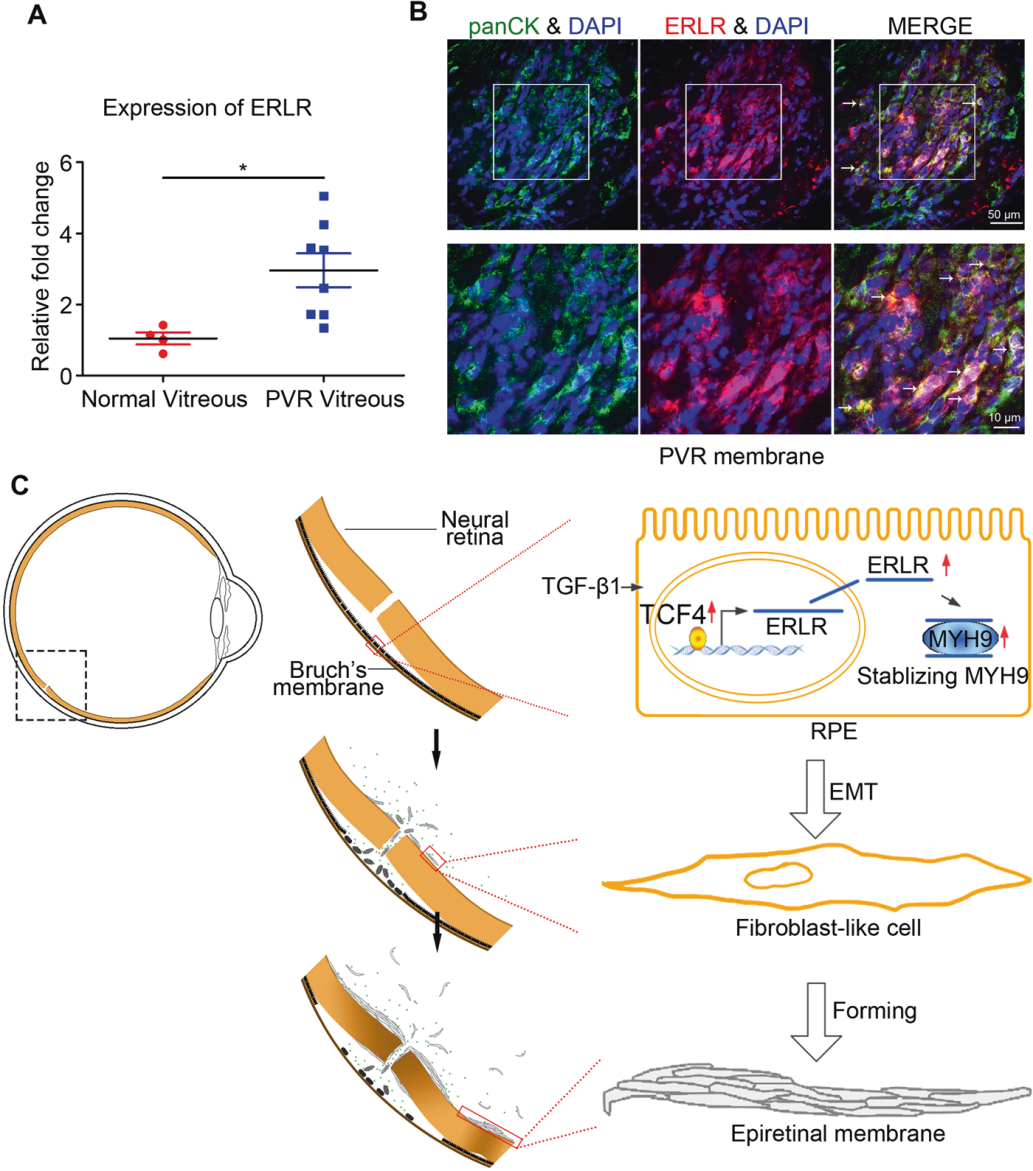
ERLR expression is associated with clinical PVR. **A** PhRPE cells were cultured with 25% PVR vitreous (*N* = 8 samples) or normal vitreous samples (*N* = 4 samples) for 48 h. ERLR expression was detected by RT-PCR. Data are presented as means ± SEM. **P < 0.01 by two-tailed Students’s *t* test. **B** PVR subretinal membranes were obtained from surgery. ERLR expression was detected by FISH. Pan-CK was detected by IF. White arrow marks the co-localization of ERLR and pan-CK. **C** A diagram depicting the mechanism of ERLR in EMT of RPE cells (modified with permission from our previous published paper in *Discovery Medicine* [3]). TGF-β1 upregulates TCF4 expression, which activates the transcription of ERLR. ERLR binds to MYH9 and increases its stability, which leads to increased MYH9 protein expression and EMT. These cells then migrate, proliferate, aggregate and form the epiretinal or subretinal membrane, which contracts and ultimately leads to tractional retinal detachment.

To assess whether ERLR is involved in the pathological progression of PVR, we measured ERLR expression in phRPE cells treated with clinical vitreous samples from PVR patients. Specifically, we assessed 4× diluted vitreous samples from clinical patients with PVR or samples from normal donor eyes for 48 h. ERLR expression was significantly upregulated in phRPE cells exposed to vitreous samples from PVR patients (Fig. 7A). We then used FISH to detect ERLR expression in clinical PVR subretinal membranes obtained through surgery. Further immunostaining of pancytokeratin (pan-CK) was performed to confirm its colocalization with ERLR. The results showed that ERLR was expressed in the PVR membrane and was partially colocalized with pan-CK (Fig. 7B), indicating its expression in RPE cells.

## Discussion

During PVR pathogenesis, RPE cells can be reprogrammed into fibrotic cells through EMT, leading to the formation of the contractile epiretinal or subretinal membrane [3, 10, 19]. Thus, the molecular mechanisms driving this conversion should be thoroughly understood. Although various lncRNAs have been revealed to play significant roles in the EMT of cancer cells [20], critical lncRNAs in the EMT of RPE cells and the underlying mechanisms are seldom reported [18, 21]. In this study, we screened the altered expression profiles of lncRNAs using lncRNA microarray analysis of RPE cells treated with TGF-β1, which is a critical cytokine expressed in vitreous samples from PVR patients and a strong EMT inducer. A total of 525 lncRNAs were significantly dysregulated, implying that lncRNAs participate in the EMT of RPE cells. We further focused on ERLR (LINC01705-201), which exhibited relatively high expression and a high fold change. RT-PCR experiments confirmed that ERLR was increased in ARPE-19 cells and phRPE cells treated with TGF-β1. However, no functional or molecular studies on ERLR have been performed to date. Thus, we characterized the full-length ERLR through RACE and revealed that ERLR is located in the cytoplasm of RPE cells. These results provide not only an enhanced understanding of the molecular characteristics of ERLR but also a basis for further functional and mechanistic studies because the function of lncRNAs strongly depends on the length and location [13, 22].

Various lncRNAs, such as MALAT-1, ATB, and HOTAIR, mediate the EMT of cancer cells through various mechanisms [23–25]. In our study, by knocking down ERLR expression via siRNA treatment and by overexpressing ERLR with a lentiviral vector, we found that ERLR is a mediator of EMT in RPE cells. In the setting of PVR, RPE cells acquire a strong ability to migrate and mediate collagen contraction, leading to the formation of fibrotic contractile membranes [26, 27]. In our study, ERLR promoted the biological functions of RPE cells, including their ability to migrate and mediate collagen gel contraction. These results strongly suggest that ERLR may play an essential role in PVR pathogenesis.

To further address how ERLR is expressed in TGF-β1 treated RPE, we explored the transcription factors involved in ERLR expression. Our data demonstrated that TCF4, a WNT signaling component capable of mediating TGF-β signaling and promoting EMT in various cancers [28, 29], directly bound to the ERLR promoter region and activated its expression.

Cytoplasmic lncRNAs can act by activating specific signaling pathways [13]. For example, the lncRNA LFAR1 promotes liver fibrosis by enhancing Smad2/3 phosphorylation [30]. Yuan et al. [24] found that lncRNA-ATB partially regulates the EMT of hepatoma cells by activating STAT3 signaling. Thus, we explored whether ERLR affects TGF-β signaling. Our study showed that ERLR inhibition does not affect the phosphorylation of Smad2/3 and P38, which are two principal pathways in TGF-β1 signaling. Cytoplasmic lncRNAs can also regulate the stability of functional proteins, thus increasing the expression of proteins [31, 32]. Therefore, we explored whether ERLR could act in this manner. RNA pull-down assays combined with MS and WB identified MHY9. In the literature, MYH9 has been described as a strong EMT inducer that promotes the progression of various cancers [33–36]. Here, our experiments confirmed that MYH9 is also expressed in RPE cells. ERLR interacts with MYH9, increasing its protein expression possibly by stabilizing it, which ultimately leads to EMT in RPE cells.

In PVR settings, RPE cells are exposed to various cytokines and growth factors as a result of the breakdown of the blood retinal barrier (BRB). Thereafter, several become dysregulated in phRPEs, thereby driving the formation of contractile membranes [37, 38]. Our studies indicated that RPE cells incubated with vitreous PVR samples expressed a high level of ERLR. Researchers found that EMT markers, such as collagen I and α-SMA, are expressed in PVR membranes [10, 19]. Our study indicated that PVR clinical membrane-derived RPE (pan-CK^+^) cells exhibit positive ERLR expression. Further in vivo studies in rabbits indicated that ERLR knockdown hampers PVR pathogenesis, alleviates traction RD, and decreases α-SMA expression. These results strongly demonstrated that ERLR is essential for PVR development. In addition, as PVR shares some common mechanisms with other fibrosis-related diseases, such as liver fibrosis or lung fibrosis, our new findings about ERLR may imply new insights for these diseases.

In conclusion, our current findings demonstrate the crucial role of ERLR in PVR pathogenesis. Our data indicate that TCF4 triggers the transcription of ERLR, which binds to and possibly stabilizes MYH9, increases its expression, and finally induces EMT in RPE cells. These cells then migrate, proliferate, aggregate and form the epiretinal or subretinal membrane, which contracts and ultimately leads to tractional retinal detachment (Fig. 7C). These results underscore the critical role of ERLR in PVR development and its potential therapeutic value.

## Supplementary information

Supplementary tables and Legends for supplementary Figures

Supplementary File 1 list of lncRNA in RPE regulated by TGF

Supplementary File 2 Proteins identification results of IP

Figure S1

Figure S2

Figure S3

Figure S4

Figure S5

Figure S6

Figure S7
