# Supplementary material for: Long noncoding RNA ERLR mediates epithelial-mesenchymal transition of retinal pigment epithelial cells and promotes experimental proliferative vitreoretinopathy
Source: Cell Death Differ. 2021 Mar 4;28(8):2351–66. doi: 10.1038/s41418-021-00756-5 (PMC8329214; doi:10.1038/s41418-021-00756-5)
Supplement: Supplementary file 1 — Supplementary tables and Legends for supplementary Figures [file 41418_2021_756_MOESM1_ESM.docx]

| Patient | gender | age | PVR stage |
| --- | --- | --- | --- |
| 1 | Male | 45 | D1 |
| 2 | Female | 39 | D3 |
| 3 | Male | 56 | D3 |
| 4 | Male | 49 | D1 |
| 5 | Female | 60 | D2 |
| 6 | Male | 58 | D1 |
| 7 | Female | 69 | D2 |
| 8 | Female | 67 | D1 |

**Supplementary Table 1.** Clinical information of PVR patients

| Donor | Gender | age | Cause of death |
| --- | --- | --- | --- |
| 1 | Male | 49 | Cerebral hemorrhage |
| 2 | Male | 71 | Myocardial infarction |
| 3 | Female | 64 | Ischemic stroke |
| 4 | Male | 34 | Ischemic shock after traffic accident |

**Supplementary Table 2.** Clinical information of Doner eyes

**Supplementary Table 3.** Sequences of siRNAs

| siRNA | Sense | Anti-sense |
| --- | --- | --- |
| ERLR-1 | GAAGAGUAGAGUGAACAAUTT | AUUGUUCACUCUACUCUUCTT |
| ERLR-2 | GCGGAACUGUUGAAAUACATT | UGUAUUUCAACAGUUCCGCTT |
| TCF4-1 | GGGACAUGCAUGGAAUCAUTT | AUGAUUCCAUGCAUGUCCCTT |
| TCF4-2 | CUCAUCGUCUCCUAAUUAUTT | AUAAUUAGGAGACGAUGAGTT |
| MYH9-1 | CGUGCGUGCUCAUGAUAAATT | UUUAUCAUGAGCACGCACGTT |
| MYH9-2 | GGACCUUCCACAUCUUCUATT | UAGAAGAUGUGGAAGGUCCTT |
| NC | UUCUCCGAACGUGUCACGUTT | ACGUGACACGUUCGGAGAATT |

**Supplementary Table 4.** Gene specific primer (GSP) and nested gene specific primer (NGSP) sequences for RACE study

| Primer | Sequences |
| --- | --- |
| 5’GSP | GATTACGCCAAGCTTGCATGAGTGGCTCTGATGCAGGA |
| 5’NGSP | GATTACGCCAAGCTTTACGATGCAACGGTGTCAGA |
| 3’GSP | GATTACGCCAAGCTTTGGGACTTGGCCTGCTGGAGGGG |
| 3’NGSP | GATTACGCCAAGCTTCCTGGCAAGAGGAGCTGGCAGGA |

**Supplementary Table 5.** Primers sequences used in RT-PCR in this study

| Primers | Forward primer | Reverse primer | Product size |
| --- | --- | --- | --- |
| ERLR | GCAAAGGATAAAGCGCTCCC | TACGATGCAACGGTGTCAGA | 134 |
| U2 | ATCGCTTCTCGGCCTTTTG | CTATTCCATCTCCCTGCTCCA | 124 |
| E-Cadherin | TCACGCTGTGTCATCCAACGG | TAGGTGTTCACATCATCGTCCGC | 192 |
| ZO-1 | AGCCATTCCCGAAGGAGTTGAG | ATCACAGTGTGGTAAGCGCAGC | 175 |
| α-SMA | CAGAAGGAGATCACGGCCCTAG | CGGCTTCATCGTATTCCTGTTTG | 157 |
| Fibronectin | AAGACCATACCCGCCGAATG | GGCATTTGGATTGAGTCCCG | 109 |
| TCF4 | GCCCAACATTCCTGCATAGCC | CATCAGCAAGCACTGCCGACT | 170 |
| MYH9 | CAGTGTTCCGCTCCTTCTT | AATACCGCTTCCTGTCCAA | 186 |
| GAPDH | CATCAGCAATGCCTCCTGCAC | TGAGTCCTTCCACGATACCAAAGTT | 86 |
| β-actin | ATCGCTTCTCGGCCTTTTG | CAGGAAGGAAGGCTGGAAGAGTG | 185 |

|  |
| --- |
|  |

**Supplementary Table 6.** siRNA sequences used in shRNA lenti-virus

|  | Target Seq |
| --- | --- |
| Lv-sh-ERLR | GAAGAGTAGAGTGAACAAT |
| Lv-sh-ERLR-2 | GCGGAACTGTTGAAATACA |
| Lv-sh-NC | TTCTCCGAACGTGTCACGT |

**Supplementary Table 7.** Primers sequences used in Chromatin immunoprecipitation (ChIP) in this study

| Primers | Forward primer | Reverse primer | Product size |
| --- | --- | --- | --- |
| ERLR promotor1 | TGGAGGAGTGGCAGATGGTTAT | GGTGAGCAGCAGTTCGTAGAC | 250 |
| ERLR promotor2 | CCCAGCAACCACACAACATAAG | AGGCTTCAATCAGGCGCTAC | 270 |
| ERLR promotor3 | GAGAGCAAGTCCTTGGTTAGTCAA | CCATCAGTGTTCCCAGACAGTATT | 115 |
| ERLR promotor4 | TGACTCATTGTCCAGATGCAGTGA | ACAGCCAAACCATATCAGTCCCATA | 199 |
| ERLR promotor5 | GGGACTGATATGGTTTGGCTGTAT | CCATCAGACCTTGTGAGGCTTAC | 154 |

**Supplementary Table 8.**Primers used for synthesizing biotin-labeled ERLR and antisense-ERLR

| Gene | Forward primer | Reverse primer |
| --- | --- | --- |
| T7-ERLR | TAATACGACTCACTATAGGGCTCTTTGCCTGCTGC | CTTGCCCGGCCATATTATTT |
| T7-antisense ERLR | TAATACGACTCACTATAGGCTTGCCCGGCCATATT | GCTCTTTGCCTGCTGCTGCA |

| **GeneSymbol** | **Expression abundance compared with GAPDH(%)** | **Fold change after TGF-β1 treatment** |
| --- | --- | --- |
| RP1-27K12.4 | 0.20 | 1.70 |
| RP11-600K15.1 | 0.036 | 2.44 |
| LOC100505718 | 0.028 | 2.98 |
| BX004987.6 | 0.060 | 2.47 |
| AK125001 | 0.12 | 2.90 |
| XLOC_007116 | 0.076 | 3.24 |
| LOC100216001 | 0.0045 | 3.57 |
| RP11-16L9.2 | 0.020 | 2.86 |
| RP11-117P22.1 | 0.0084 | 4.58 |
| RP1-272L16.1 | 0.0035 | 3.85 |
| AC104820.2 | 0.0067 | 2.31 |
| MYH16 | 0.14 | 4.35 |
| XLOC_006210 | 0.027 | 3.28 |
| RP1-272L16.1 | 0.096 | 2.32 |
| RP11-99L13.2 | 0.0081 | **4.98** |
| **RP11-400N13.3** | **0.21** | **4.65** |
| chr7:46184850-46198900 | 0.028 | 3.15 |
| uc.87 | 0.074 | 3.60 |
| XLOC_008704 | 0.023 | **4.74** |
| KB-1000E4.2 | 0.11 | 3.51 |

**Supplementary Table 9. Expression of selected LncRNAs in phRPE confirmed by RT-PCR**

**Supplementary Figure legends**

**Supplementary Figure 1.** Determining the full length of ERLR by RACE. A. Total RNA was extracted from phRPE cells. Nested RACE was performed using a SMARTer™ RACE cDNA amplification kit (Clontech) according to the manufacturer’s instructions. A. The RACE products were first characterized by agarose gel electrophoresis. After the RACE products were cloned, the plasmids containing these products were sequenced. The initiation and termination regions of the sequencing results are shown. B–D. Full-length ERLR sequence data were obtained from the sequencing of the RACE products. Heterogeneity of the 3′-end of ERLR was revealed. Three different ends of ERLR are shown. Bases shown in green and blue are gene-specific and nested gene-specific primer sites, respectively. Bases presented in red are new bases determined by our RACE sequencing. The yellow highlighted bases are different bases across three ERLR transcript variants.

**Supplementary** Figure 2. Coding potential and conservative information about ERLR. A-C: Predicting the coding potential of ERLR by ORF Finder (A), Coding Potential Calculator (B), and Coding-Potential Assessment Tool (C). D: UCSC Genome Browser information on ERLR.

**Supplementary Figure 3. ERLR shRNA-lentiviruses (lv-sh-ERLR and lv-sh-ERLR-2) transfection significantly inhibited ERLR expression in phRPE cells.** phRPE cells were transfected with lv-sh-ERLR, lv-sh-ERLR-2 or the negative control shRNA lentivirus (lv-sh-NC) for 48 h. RT-PCR was conducted to detect ERLR expression in phRPE cells. (N=3 independent experiments/group. Each dot represents the average of 3 technical replicates from a single independent experiment). Data are presented as means ± SEM. *P<0.05 by two tailed Students’s t-test.

**Supplementary Figure 4.** Knocking down ERLR by another shRNA lentivirus (lv-sh-ERLR-2) also hampers the PVR initiating ability of phRPE cells in an experimental PVR model. PhRPE cells were transfected with lv-sh-NC or lv-sh-ERLR-2 and then injected into the vitreous body of pigmented rabbits to induce PVR. A. PVR severity in each group was graded according to Fastenberg’s score at the indicated times. N=6 rabbits. Data are presented as means ± SEM.NS: not significant, *P < 0.05 by Mann-Whitney U test. B. Fundus photographs in each group were captured at the indicated times by a smartphone under a microscope with the help of a Volk SuperQuad 160 fundus lens. Localized detachment of medullary ray is shown in eyes of Lv-sh-NC group at day 21 (red arrow). Total RD with retinal folds and holes (red triangle) is shown at day 28. Focal traction and detachment (red arrow) is shown in the eyes of Lv-sh-ERLR-2 group at day 28. C. Ultrasound B scanning was performed at day 28. D. H&E staining of the rabbit eye in each group was performed and analyzed. A representative image is shown. Bar=50 μm. E. α-SMA expression around the retina of the rabbits was detected by immunofluorescence. The white arrow marks the α-SMA-positive epiretinal membrane. Representative results are shown.

**Supplementary Figure 5. Screening process to identify transcription factors (TFs) associated with ERLR.** A. Identification of the TFs predicted to associate with the promoter of ERLR, as determined by PROMO software. A total of 56 transcription factors were identified. B. The upregulated TFs according to the microarray results of ARPE-19 cells treated with or without TGF-β1 are presented. C. Regression analysis was conducted to determine the relationship between TCF4 and ERLR based on the microarray results. D. TCF4 expression in phRPE cells treated with or without TGF-β1 was detected by qRT-PCR. Regression analysis revealed a positive correlation between ERLR and TCF4 expression. E. The primer pairs recognized different regions within the ERLR promoter.

**Supplementary Figure 6. Coding and non-coding gene (CNC) network of genes of interest.**

**Supplementary Figure 7. Modulating ERLR expression had no effect on Smad2/3 and P38 activities.** A. PhRPE cells transfected with Si-ERLR-1 or Si-NC were treated with TGF-β1 (10 ng/ml) for 1 h. A. Expression levels of p-Smad2/3 and Smad2/3 (A), p-38, and p38 (B) were examined by WB.
