# Supplementary material for: Long noncoding RNA ERLR mediates epithelial-mesenchymal transition of retinal pigment epithelial cells and promotes experimental proliferative vitreoretinopathy
Source: Cell Death Differ. 2021 Mar 4;28(8):2351–66. doi: 10.1038/s41418-021-00756-5 (PMC8329214; doi:10.1038/s41418-021-00756-5)
Supplement: Supplementary file 2 — Supplementary File 1 list of lncRNA in RPE regulated by TGF [file 41418_2021_756_MOESM2_ESM.docx]

| **Supplementary file 1**, related to Figure 1. List of lncRNAs in RPE cells regulated by TGF-β1 | | | | | | |
| --- | --- | --- | --- | --- | --- | --- |
| **ProbeName** | **P-value** | **Fold Change** | **Regulation** | **seqname** | **GeneSymbol** |  |
| ASHGA5P042470 | 0.002306403 | 12.934535 | up | ENST00000508884 | RP1-27K12.4 |  |
| ASHGA5P044318 | 0.00078323 | 11.524944 | up | ENST00000519062 | RP11-600K15.1 |  |
| ASHGA5P044317 | 0.001236417 | 9.5303088 | up | NR_039986 | LOC100505718 |  |
| ASHGA5P040190 | 0.001233069 | 8.0916549 | up | ENST00000429884 | BX004987.6 |  |
| ASHGA5P016889 | 0.003574616 | 8.0480427 | up | uc002uns.1 | AK125001 |  |
| ASHGA5P046928 | 0.001723371 | 7.2060867 | up | TCONS_00015035 | XLOC_007116 |  |
| ASHGA5P045869 | 0.000140564 | 6.7985977 | up | uc001ihe.4 | LOC100216001 |  |
| ASHGA5P043370 | 5.63735E-07 | 5.7724424 | up | ENST00000422480 | RP11-16L9.2 |  |
| ASHGA5P017633 | 0.001703868 | 5.5533543 | up | ENST00000443994 | RP11-117P22.1 |  |
| ASHGA5P054239 | 2.43956E-05 | 5.0092634 | up | ENST00000453331 | RP1-272L16.1 |  |
| ASHGA5P036289 | 0.003809168 | 4.876869 | up | ENST00000428474 | AC104820.2 |  |
| ASHGA5P043801 | 4.11444E-05 | 4.6606977 | up | ENST00000453378 | MYH16 |  |
| ASHGA5P049587 | 0.006582745 | 4.6444325 | up | TCONS_00013565 | XLOC_006210 |  |
| ASHGA5P032437 | 0.001248747 | 4.5809578 | up | ENST00000424696 | RP1-272L16.1 |  |
| ASHGA5P055977 | 0.020126266 | 4.4788112 | up | ENST00000557721 | RP11-99L13.2 |  |
| **ASHGA5P032711** | **0.005265916** | **4.2429853** | **up** | **ENST00000438158** | **RP11-400N13.3** |  |
| ASHGA5P000504 | 0.006984479 | 4.2265414 | up | chr7:46184850-46198900+ | chr7:46184850-46198900 |  |
| ASHGA5P058713 | 0.000478544 | 4.0256651 | up | uc.87- | uc.87 |  |
| ASHGA5P044446 | 0.009713916 | 4.0187672 | up | TCONS_00018417 | XLOC_008704 |  |
| ASHGA5P044424 | 0.005334849 | 4.0051044 | up | ENST00000569849 | KB-1000E4.2 |  |
| ASHGA5P032432 | 0.008667377 | 4.0003827 | up | ENST00000416349 | RP11-385M4.1 |  |
| ASHGA5P043834 | 0.000619377 | 3.9469652 | up | ENST00000420058 | RP11-645N11.2 |  |
| ASHGA5P027324 | 0.000793702 | 3.9304054 | up | ENST00000508763 | RP11-834C11.5 |  |
| ASHGA5P044200 | 0.001098045 | 3.8315365 | up | ENST00000520431 | RP11-527N22.2 |  |
| ASHGA5P047200 | 0.034970965 | 3.8272717 | up | ENST00000583117 | LINC00200 |  |
| ASHGA5P028542 | 0.007382985 | 3.7694156 | up | NR_046414 | LINC00441 |  |
| ASHGA5P053200 | 0.011406149 | 3.7244538 | up | ENST00000452010 | MYH16 |  |
| ASHGA5P029100 | 0.013235524 | 3.6741289 | up | ENST00000557506 | RP11-99L13.2 |  |
| ASHGA5P026060 | 0.014489767 | 3.6481642 | up | ENST00000419440 | AP004372.1 |  |
| ASHGA5P038212 | 0.006855369 | 3.6401879 | up | TCONS_00014864 | XLOC_006933 |  |
| ASHGA5P020826 | 1.51965E-05 | 3.5449831 | up | ENST00000520422 | RP11-150O12.1 |  |
| ASHGA5P057291 | 5.27005E-05 | 3.5232615 | up | TCONS_00015426 | XLOC_007053 |  |
| ASHGA5P027634 | 0.010042961 | 3.4967865 | up | ENST00000561881 | RP11-490G2.2 |  |
| ASHGA5P043401 | 0.000122232 | 3.3974888 | up | ENST00000427825 | RP11-469A15.2 |  |
| ASHGA5P054618 | 0.001167143 | 3.3726751 | up | ENST00000522718 | RP11-150O12.1 |  |
| ASHGA5P031805 | 0.028890045 | 3.3704472 | up | TCONS_00007179 | XLOC_003148 |  |
| ASHGA5P020910 | 0.001908392 | 3.3572167 | up | ENST00000521870 | CTC-558O2.1 |  |
| ASHGA5P029935 | 0.003103235 | 3.2389214 | up | ENST00000560743 | RP11-265N7.1 |  |
| ASHGA5P053704 | 0.000958806 | 3.1675651 | up | NR_046175 | LOC286297 |  |
| ASHGA5P017901 | 0.037946115 | 3.1422104 | up | ENST00000446952 | RP11-528G1.2 |  |
| ASHGA5P040846 | 0.000570042 | 3.13398 | up | TCONS_00021754 | XLOC_010348 |  |
| ASHGA5P057222 | 0.002661859 | 3.1105448 | up | TCONS_00014746 | XLOC_006830 |  |
| ASHGA5P045365 | 0.026112425 | 2.9882202 | up | ENST00000455051 | RP11-341A22.2 |  |
| ASHGA5P056017 | 0.008613108 | 2.9746617 | up | NR_039985 | FLJ22447 |  |
| ASHGA5P057117 | 0.00147577 | 2.9634335 | up | TCONS_00013473 | XLOC_006121 |  |
| ASHGA5P045870 | 0.002262901 | 2.9590351 | up | NR_024475 | LOC100216001 |  |
| ASHGA5P015325 | 0.005225864 | 2.9061567 | up | ENST00000419264 | AC113617.1 |  |
| ASHGA5P043690 | 0.017726907 | 2.9045995 | up | ENST00000442436 | RP11-321E8.4 |  |
| ASHGA5P023575 | 0.000963423 | 2.8966436 | up | ENST00000584612 | KRT16P2 |  |
| ASHGA5P037957 | 0.010289105 | 2.8498422 | up | ENST00000453924 | SC22CB-1D7.1 |  |
| ASHGA5P054902 | 0.001352878 | 2.812053 | up | TCONS_00008628 | XLOC_004134 |  |
| ASHGA5P039924 | 0.000807257 | 2.8099688 | up | ENST00000425449 | RP4-663N10.1 |  |
| ASHGA5P054465 | 0.014786249 | 2.8044461 | up | ENST00000435813 | RP11-346D6.6 |  |
| ASHGA5P040074 | 0.003080895 | 2.7934235 | up | ENST00000420701 | AC113617.1 |  |
| ASHGA5P056846 | 0.010902913 | 2.7871679 | up | TCONS_00009842 | XLOC_004256 |  |
| ASHGA5P040075 | 0.006375353 | 2.7781792 | up | ENST00000448804 | AC113617.1 |  |
| ASHGA5P040076 | 0.020564446 | 2.7687798 | up | ENST00000426240 | AC113617.1 |  |
| ASHGA5P055012 | 0.02918455 | 2.7425405 | up | ENST00000531320 | ZNF705E |  |
| ASHGA5P039503 | 0.015891261 | 2.7398157 | up | ENST00000510795 | RP11-168E14.1 |  |
| ASHGA5P017617 | 0.004821396 | 2.7062614 | up | ENST00000443778 | LINC00327 |  |
| ASHGA5P037758 | 1.26025E-05 | 2.6761918 | up | uc010ofi.1 | SFN |  |
| ASHGA5P035828 | 0.020939086 | 2.6645027 | up | ENST00000444629 | AC007254.3 |  |
| ASHGA5P054351 | 0.000643629 | 2.6450564 | up | ENST00000449712 | RP11-117P22.1 |  |
| ASHGA5P017143 | 0.000118917 | 2.6318991 | up | NR_038949 | LOC100506195 |  |
| ASHGA5P025359 | 0.000897548 | 2.6127426 | up | NR_002785 | GNAS-AS1 |  |
| ASHGA5P026547 | 0.025761647 | 2.5760085 | up | AK094629 |  |  |
| ASHGA5P021540 | 0.006436868 | 2.5703987 | up | ENST00000534431 | RP4-541C22.5 |  |
| ASHGA5P030116 | 0.005222279 | 2.521436 | up | ENST00000559495 | RP11-809H16.4 |  |
| ASHGA5P039716 | 0.022411367 | 2.4873247 | up | ENST00000512547 | RP11-665C14.2 |  |
| ASHGA5P057731 | 0.011604688 | 2.483388 | up | TCONS_00021269 | XLOC_009974 |  |
| ASHGA5P036129 | 0.002220301 | 2.4552116 | up | ENST00000419223 | AC023347.1 |  |
| ASHGA5P015867 | 0.00174393 | 2.4498338 | up | ENST00000448275 | CTA-282F2.3 |  |
| ASHGA5P028616 | 0.000553327 | 2.4382595 | up | ENST00000423249 | BX004987.4 |  |
| ASHGA5P038081 | 0.019182575 | 2.4370553 | up | NR_004428 | EGOT |  |
| ASHGA5P049829 | 0.031521041 | 2.4273905 | up | ENST00000584749 | LINC00511 |  |
| ASHGA5P023479 | 0.028846767 | 2.4237641 | up | ENST00000581801 | LINC00511 |  |
| ASHGA5P031117 | 0.012192409 | 2.3921801 | up | ENST00000561541 | RP11-19N8.4 |  |
| ASHGA5P019641 | 0.009441818 | 2.3881902 | up | NR_052005 | CD274 |  |
| ASHGA5P030686 | 0.010409086 | 2.3562261 | up | TCONS_00006337 | XLOC_002952 |  |
| ASHGA5P044316 | 0.009169197 | 2.3517568 | up | ENST00000522354 | RP11-865I6.2 |  |
| ASHGA5P039015 | 0.004589205 | 2.322951 | up | ENST00000463373 | RP11-39E4.1 |  |
| ASHGA5P034605 | 0.047514283 | 2.318674 | up | ENST00000443870 | LOC147976 |  |
| ASHGA5P056349 | 0.010010737 | 2.3128733 | up | TCONS_00003617 | XLOC_001406 |  |
| ASHGA5P028557 | 0.015803081 | 2.3025357 | up | ENST00000415979 | AL583842.2 |  |
| ASHGA5P055544 | 0.009701953 | 2.2832528 | up | ENST00000550380 | RP11-54A9.1 |  |
| ASHGA5P019778 | 2.31995E-05 | 2.280143 | up | ENST00000503066 | RP11-291L15.2 |  |
| ASHGA5P022726 | 7.35341E-05 | 2.2727481 | up | ENST00000561588 | RP11-211G23.2 |  |
| ASHGA5P016775 | 0.00046751 | 2.2713864 | up | ENST00000434150 | RP4-758J18.10 |  |
| ASHGA5P040748 | 0.000183567 | 2.2688774 | up | TCONS_00022133 | XLOC_010743 |  |
| ASHGA5P044167 | 0.000156843 | 2.2653564 | up | TCONS_00018504 | XLOC_008812 |  |
| ASHGA5P032659 | 0.003534121 | 2.2625607 | up | TCONS_00029162 | XLOC_014106 |  |
| ASHGA5P045274 | 0.048491758 | 2.238923 | up | ENST00000414515 | RP11-195E11.2 |  |
| ASHGA5P039719 | 0.010180688 | 2.2372677 | up | ENST00000505537 | AC108142.1 |  |
| ASHGA5P019924 | 0.004247281 | 2.2315945 | up | ENST00000505339 | CTD-2066L21.3 |  |
| ASHGA5P025896 | 0.000128811 | 2.2289083 | up | NR_046072 | NKX3-1 |  |
| ASHGA5P049894 | 0.007540843 | 2.2197628 | up | TCONS_00001243 | XLOC_000523 |  |
| ASHGA5P019188 | 0.017136714 | 2.2167398 | up | ENST00000472193 | PLCL1 |  |
| ASHGA5P017081 | 1.93512E-05 | 2.2095606 | up | ENST00000437488 | RP11-385J1.2 |  |
| ASHGA5P034765 | 0.028439289 | 2.1955752 | up | TCONS_00015918 | XLOC_007281 |  |
| ASHGA5P041267 | 0.008687437 | 2.1864125 | up | ENST00000513288 | RP11-455B3.1 |  |
| ASHGA5P007321 | 0.008944236 | 2.1855489 | up | ENST00000331369 | FUNDC2P2 |  |
| ASHGA5P053719 | 0.010255861 | 2.1774619 | up | NR_040076 | LOC100507244 |  |
| ASHGA5P017063 | 0.00088957 | 2.1634978 | up | ENST00000437232 | RP11-124N14.4 |  |
| ASHGA5P018162 | 0.013866624 | 2.1573654 | up | NR_034126 | LOC100129269 |  |
| ASHGA5P045179 | 0.019385299 | 2.1560665 | up | uc003zqt.3 | BX538226 |  |
| ASHGA5P028153 | 0.002416677 | 2.1531368 | up | ENST00000553247 | RP11-54A9.1 |  |
| ASHGA5P057208 | 0.001566254 | 2.1460554 | up | TCONS_00014617 | XLOC_006712 |  |
| ASHGA5P035356 | 0.005508139 | 2.1271841 | up | ENST00000441356 | AC104777.2 |  |
| ASHGA5P058337 | 0.000341831 | 2.1201818 | up | uc001iot.1 | BC078172 |  |
| ASHGA5P037130 | 0.002672628 | 2.1076812 | up | uc002xyu.1 | AK056098 |  |
| ASHGA5P045865 | 0.011121507 | 2.1033949 | up | ENST00000454470 | RP11-67B16.1 |  |
| ASHGA5P022472 | 0.029631674 | 2.0969867 | up | ENST00000557544 | RP11-618G20.4 |  |
| ASHGA5P038519 | 0.022003628 | 2.0912178 | up | ENST00000486545 | RP11-439C8.2 |  |
| ASHGA5P027730 | 0.017072783 | 2.0898891 | up | uc001qjg.1 | AX746535 |  |
| ASHGA5P023451 | 0.002695864 | 2.0875844 | up | ENST00000581210 | KRT16P1 |  |
| ASHGA5P029782 | 0.026191757 | 2.0874125 | up | ENST00000427085 | AL132709.5 |  |
| ASHGA5P028502 | 0.001807635 | 2.0863762 | up | TCONS_00010589 | XLOC_005115 |  |
| ASHGA5P019580 | 0.029712367 | 2.0776943 | up | ENST00000493529 | RP11-298O21.5 |  |
| ASHGA5P040117 | 0.007810474 | 2.0764969 | up | ENST00000507332 | RP11-614F17.2 |  |
| ASHGA5P025887 | 0.005971173 | 2.0722771 | up | NR_045669 | PSMC3IP |  |
| ASHGA5P000374 | 0.008810335 | 2.0667392 | up | chr2:90959050-90986900+ | chr2:90959050-90986900 |  |
| ASHGA5P026011 | 0.000259576 | 2.0628582 | up | NR_072994 | PPP1R10 |  |
| ASHGA5P050448 | 0.00219686 | 2.0551933 | up | ENST00000418854 | AC007254.3 |  |
| ASHGA5P015068 | 0.001976465 | 2.0457493 | up | ENST00000416275 | CTA-282F2.3 |  |
| ASHGA5P040384 | 0.038937062 | 2.0381779 | up | ENST00000511840 | CTD-2066L21.2 |  |
| ASHGA5P023476 | 0.021780172 | 2.0271155 | up | ENST00000581690 | RP11-805F19.1 |  |
| ASHGA5P019253 | 0.005251785 | 2.0248855 | up | ENST00000475362 | RP11-631B21.1 |  |
| ASHGA5P038527 | 0.004752088 | 2.0183374 | up | TCONS_00023979 | XLOC_011308 |  |
| ASHGA5P041206 | 0.005798309 | 2.0159693 | up | ENST00000514696 | CTD-2269F5.1 |  |
| ASHGA5P026341 | 0.002168715 | 2.0114185 | up | ENST00000534604 | RP11-430H10.3 |  |
| ASHGA5P044356 | 0.000184531 | 2.0034219 | up | ENST00000523871 | RP11-48B3.3 |  |
| ASHGA5P009370 | 0.002864606 | 2.0017969 | up | ENST00000354642 | RP11-573D15.8 |  |
| ASHGA5P044668 | 0.004968193 | 2.0014722 | up | ENST00000521055 | RP11-13N12.2 |  |
| ASHGA5P042824 | 0.006719944 | 16.623475 | down | TCONS_00019778 | XLOC_009577 |  |
| ASHGA5P018965 | 0.000225381 | 13.063422 | down | ENST00000462528 | RP11-768G7.2 |  |
| ASHGA5P056665 | 0.000179213 | 12.808042 | down | TCONS_00007014 | XLOC_002888 |  |
| ASHGA5P016523 | 3.09869E-06 | 12.293453 | down | ENST00000431296 | LINC00202 |  |
| ASHGA5P034032 | 0.036309189 | 12.064329 | down | ENST00000326734 | FAM87B |  |
| ASHGA5P034649 | 1.44078E-05 | 11.374163 | down | ENST00000425982 | AC005392.13 |  |
| ASHGA5P034650 | 1.26589E-05 | 11.352108 | down | NR_026881 | PRG1 |  |
| ASHGA5P040095 | 0.000416444 | 11.153995 | down | uc003iet.3 | BC045668 |  |
| ASHGA5P019538 | 0.048360786 | 10.641899 | down | uc003feg.3 | BC073807 |  |
| ASHGA5P056111 | 1.37076E-05 | 10.633572 | down | TCONS_00026975 | XLOC_013026 |  |
| ASHGA5P027409 | 3.36496E-05 | 10.526883 | down | uc001stu.3 | BC035381 |  |
| ASHGA5P015360 | 1.59791E-06 | 9.7768668 | down | ENST00000419624 | AC005392.13 |  |
| ASHGA5P033681 | 0.012852759 | 8.521828 | down | ENST00000575722 | RP11-849I19.1 |  |
| ASHGA5P053005 | 0.00042487 | 8.5068937 | down | uc003syl.3 | BC035889 |  |
| ASHGA5P023084 | 0.000227533 | 8.140846 | down | ENST00000569328 | RP11-700H13.1 |  |
| ASHGA5P019877 | 0.022860447 | 8.0825512 | down | ENST00000504516 | AP005901.1 |  |
| ASHGA5P036476 | 0.002550889 | 7.466331 | down | ENST00000424395 | AC093843.1 |  |
| ASHGA5P035708 | 0.025765098 | 7.1198478 | down | ENST00000483023 | RP11-521D12.2 |  |
| ASHGA5P016443 | 0.005382279 | 6.8410446 | down | ENST00000430464 | RP11-180I22.2 |  |
| ASHGA5P017793 | 0.002913842 | 6.7467707 | down | uc001hqv.3 | BC039356 |  |
| ASHGA5P015997 | 0.003885318 | 6.5154797 | down | NR_036584 | LOC100289650 |  |
| ASHGA5P018666 | 0.001510876 | 6.4604129 | down | ENST00000456327 | AC073479.1 |  |
| ASHGA5P039135 | 0.013859759 | 6.4324641 | down | AA757150 |  |  |
| ASHGA5P030496 | 0.048949365 | 6.3888011 | down | uc001zqm.3 | BC037861 |  |
| ASHGA5P051569 | 5.16193E-05 | 6.0752664 | down | uc011bfh.2 | ID2B |  |
| ASHGA5P045003 | 0.00014718 | 6.0512726 | down | ENST00000517482 | RP11-383J24.1 |  |
| ASHGA5P049955 | 0.001093022 | 6.0446843 | down | ENST00000583081 | RP11-143J12.2 |  |
| ASHGA5P056005 | 0.001945395 | 5.9280718 | down | NR_026797 | LINC00520 |  |
| ASHGA5P053535 | 0.024377201 | 5.8867676 | down | ENST00000517308 | RP11-58O3.2 |  |
| ASHGA5P056435 | 0.023690653 | 5.8533748 | down | TCONS_00004261 | XLOC_002102 |  |
| ASHGA5P035750 | 0.001679367 | 5.7057753 | down | ENST00000435237 | AC067959.1 |  |
| ASHGA5P047029 | 0.045393725 | 5.6173317 | down | ENST00000427379 | RP11-459F3.3 |  |
| ASHGA5P040721 | 0.012136416 | 5.5909292 | down | TCONS_00022115 | XLOC_010723 |  |
| ASHGA5P027143 | 0.000294673 | 5.5885277 | down | TCONS_00011669 | XLOC_005150 |  |
| ASHGA5P042735 | 0.016144061 | 5.5714402 | down | ENST00000439207 | RP11-568A7.2 |  |
| ASHGA5P021433 | 0.015539268 | 5.5272907 | down | ENST00000532307 | CTD-2210P24.1 |  |
| ASHGA5P033469 | 0.01012953 | 5.5197045 | down | ENST00000581719 | RP11-672L10.2 |  |
| ASHGA5P039582 | 0.026338106 | 5.4830487 | down | TCONS_00022469 | XLOC_010798 |  |
| ASHGA5P032744 | 0.000159784 | 5.4332722 | down | uc002frx.1 | AX748314 |  |
| ASHGA5P033772 | 0.006221707 | 5.4056028 | down | ENST00000581351 | RP11-527H14.4 |  |
| ASHGA5P036923 | 0.009252979 | 5.3402216 | down | ENST00000418255 | RP1-118J21.5 |  |
| ASHGA5P000072 | 0.000275285 | 5.3050866 | down | AL512723 |  |  |
| ASHGA5P028760 | 0.003314348 | 5.2808817 | down | uc001utv.3 | DKFZp666K117 |  |
| ASHGA5P050508 | 0.023384582 | 5.1989106 | down | ENST00000421843 | PCBP1-AS1 |  |
| ASHGA5P017560 | 0.005061038 | 5.197285 | down | ENST00000443123 | AC006227.1 |  |
| ASHGA5P020645 | 0.041957839 | 5.1821229 | down | ENST00000517916 | CTB-54I1.1 |  |
| ASHGA5P029269 | 0.001947153 | 5.1274553 | down | ENST00000555001 | RP11-299L17.3 |  |
| ASHGA5P028761 | 0.000395507 | 5.1256723 | down | ENST00000441659 | RP11-207N4.3 |  |
| ASHGA5P056308 | 0.015931218 | 5.1171661 | down | TCONS_00002106 | XLOC_000318 |  |
| ASHGA5P054367 | 0.00392874 | 5.0791102 | down | ENST00000434919 | SFTA1P |  |
| ASHGA5P042096 | 0.000194036 | 5.0043582 | down | ENST00000455011 | RP3-460G2.2 |  |
| ASHGA5P044735 | 0.010460114 | 4.9882219 | down | ENST00000560714 | CTD-2647L4.5 |  |
| ASHGA5P030810 | 0.000832892 | 4.9200564 | down | uc021sxs.1 | AF198444 |  |
| ASHGA5P027837 | 0.002257048 | 4.8452008 | down | ENST00000538231 | RP11-392P7.6 |  |
| ASHGA5P035242 | 0.030126097 | 4.8262541 | down | TCONS_00016565 | XLOC_007326 |  |
| ASHGA5P018450 | 0.006279512 | 4.754063 | down | ENST00000453714 | AC012370.3 |  |
| ASHGA5P026721 | 0.004273481 | 4.7123706 | down | ENST00000562245 | RP11-660M18.2 |  |
| ASHGA5P007457 | 0.024495158 | 4.652188 | down | ENST00000445745 | AC016683.6 |  |
| ASHGA5P020838 | 0.016193067 | 4.6435702 | down | ENST00000520594 | RP11-152P17.2 |  |
| ASHGA5P043692 | 0.049978545 | 4.528786 | down | TCONS_00017795 | XLOC_008405 |  |
| ASHGA5P031885 | 0.014729437 | 4.5078052 | down | ENST00000562507 | RP11-394B2.4 |  |
| ASHGA5P030373 | 0.033714441 | 4.452651 | down | ENST00000428641 | RP11-66D17.5 |  |
| ASHGA5P040507 | 0.03598251 | 4.4412612 | down | ENST00000503568 | RP11-229C3.2 |  |
| ASHGA5P029949 | 0.016415373 | 4.4081171 | down | ENST00000563217 | RP11-532F12.5 |  |
| ASHGA5P000087 | 0.045626183 | 4.3937695 | down | AY726569 |  |  |
| ASHGA5P036492 | 0.030813226 | 4.3598414 | down | TCONS_00026386 | XLOC_012736 |  |
| ASHGA5P058349 | 0.000184029 | 4.3578339 | down | uc001mie.3 | BC073899 |  |
| ASHGA5P042651 | 0.016696651 | 4.35658 | down | uc003qgn.3 | LINC00271 |  |
| ASHGA5P044340 | 0.030385374 | 4.3534648 | down | ENST00000521872 | CTD-2320G14.2 |  |
| ASHGA5P021158 | 0.000360338 | 4.3407835 | down | ENST00000526617 | RP11-540A21.3 |  |
| ASHGA5P020781 | 0.004539651 | 4.2264001 | down | ENST00000519753 | CTD-3107M8.4 |  |
| ASHGA5P058029 | 0.020414915 | 4.2180699 | down | TCONS_00025507 | XLOC_012317 |  |
| ASHGA5P025913 | 0.014740266 | 4.1782647 | down | NR_046226 | CPB2-AS1 |  |
| ASHGA5P033510 | 0.000220276 | 4.1643442 | down | ENST00000579368 | RP11-674N23.1 |  |
| ASHGA5P058334 | 2.05461E-06 | 4.1441398 | down | uc001gzl.3 | BC034684 |  |
| ASHGA5P053526 | 0.001412974 | 4.1196368 | down | ENST00000524045 | RP11-152P17.2 |  |
| ASHGA5P043563 | 0.027480363 | 4.1166758 | down | TCONS_00018815 | XLOC_008609 |  |
| ASHGA5P043511 | 0.013277114 | 4.0962408 | down | ENST00000449931 | AC006042.6 |  |
| ASHGA5P020282 | 0.016581268 | 4.0630821 | down | ENST00000510859 | AC016683.6 |  |
| ASHGA5P031324 | 0.003354147 | 4.0308481 | down | ENST00000568819 | RP11-525K10.3 |  |
| ASHGA5P043030 | 0.020741085 | 4.0291125 | down | NR_038276 | LOC100506895 |  |
| ASHGA5P045953 | 0.018747681 | 3.9609392 | down | ENST00000561961 | RP11-355O1.11 |  |
| ASHGA5P041077 | 0.035794349 | 3.9534297 | down | ENST00000511712 | RP11-53O19.2 |  |
| ASHGA5P057548 | 0.002811678 | 3.9210372 | down | TCONS_00018703 | XLOC_008434 |  |
| ASHGA5P039518 | 0.044027088 | 3.9058611 | down | TCONS_00022431 | XLOC_010769 |  |
| ASHGA5P017392 | 0.047544253 | 3.9048419 | down | ENST00000440947 | RP11-120J1.1 |  |
| ASHGA5P017930 | 0.019492636 | 3.9033092 | down | ENST00000447277 | RP11-626E13.1 |  |
| ASHGA5P039243 | 0.000298311 | 3.8962554 | down | ENST00000437597 | AC046143.7 |  |
| ASHGA5P030436 | 0.001760613 | 3.8788133 | down | NR_038251 | ATPBD4-AS1 |  |
| ASHGA5P038633 | 0.00571633 | 3.8686713 | down | ENST00000455557 | AC108676.1 |  |
| ASHGA5P030582 | 0.020117182 | 3.8454545 | down | uc002ahe.3 | BC028842 |  |
| ASHGA5P029029 | 0.014905277 | 3.833799 | down | TCONS_00010324 | XLOC_004806 |  |
| ASHGA5P014572 | 6.9897E-05 | 3.8289863 | down | ENST00000442550 | LINC00113 |  |
| ASHGA5P054045 | 0.000364526 | 3.814858 | down | ENST00000420327 | RP13-348B13.2 |  |
| ASHGA5P030337 | 0.001082347 | 3.7925579 | down | TCONS_00008667 | XLOC_004182 |  |
| ASHGA5P029146 | 0.000685747 | 3.7769814 | down | ENST00000554221 | LINC00520 |  |
| ASHGA5P045062 | 0.047742773 | 3.765306 | down | ENST00000520606 | RP11-10J21.5 |  |
| ASHGA5P044674 | 0.01492957 | 3.7575279 | down | ENST00000517454 | RP11-326L2.1 |  |
| ASHGA5P022461 | 0.034801234 | 3.7502626 | down | ENST00000557242 | RP11-1017G21.4 |  |
| ASHGA5P043269 | 0.011604747 | 3.7130185 | down | ENST00000454538 | RP11-563D10.1 |  |
| ASHGA5P055076 | 0.000467562 | 3.6806524 | down | uc021qoq.1 | HP11113 |  |
| ASHGA5P033991 | 1.6215E-06 | 3.6757172 | down | TCONS_00004031 | XLOC_001869 |  |
| ASHGA5P015634 | 0.047672218 | 3.6447604 | down | ENST00000422117 | AC124997.1 |  |
| ASHGA5P026720 | 0.000289726 | 3.6331535 | down | ENST00000563681 | RP11-660M18.2 |  |
| ASHGA5P022644 | 0.001809744 | 3.6309508 | down | ENST00000560267 | LINC00520 |  |
| ASHGA5P027386 | 0.000134528 | 3.624464 | down | uc001srm.3 | AK024134 |  |
| ASHGA5P040763 | 0.008830344 | 3.6243459 | down | TCONS_00021712 | XLOC_010296 |  |
| ASHGA5P048414 | 0.000236457 | 3.5875269 | down | ENST00000530264 | RP11-693N9.2 |  |
| ASHGA5P036333 | 0.002859808 | 3.5596846 | down | TCONS_00026598 | XLOC_012624 |  |
| ASHGA5P027467 | 0.000433554 | 3.5563303 | down | uc001szx.3 | BC045559 |  |
| ASHGA5P054720 | 0.028702188 | 3.543617 | down | NR_036485 | SBF2-AS1 |  |
| ASHGA5P028476 | 0.000216136 | 3.5320003 | down | uc001utw.3 | BC035084 |  |
| ASHGA5P037796 | 0.03378717 | 3.5185322 | down | NR_026651 | DGCR10 |  |
| ASHGA5P030435 | 0.00479337 | 3.5066057 | down | ENST00000560866 | RP11-702M1.1 |  |
| ASHGA5P045799 | 0.013798214 | 3.4916812 | down | ENST00000431813 | DAPK1-IT1 |  |
| ASHGA5P058579 | 0.033693141 | 3.4825614 | down | uc010mpn.1 | PCA3 |  |
| ASHGA5P017903 | 0.000210956 | 3.4824344 | down | ENST00000446964 | RP11-1L9.1 |  |
| ASHGA5P035817 | 0.000872037 | 3.4280492 | down | NR_027252 | CYP1B1-AS1 |  |
| ASHGA5P055749 | 0.005864742 | 3.3987137 | down | ENST00000490410 | EEF1DP3 |  |
| ASHGA5P045410 | 0.048195558 | 3.3814478 | down | NR_024376 | FAM225B |  |
| ASHGA5P022491 | 0.001252542 | 3.3470686 | down | NR_027654 | SMAD6 |  |
| ASHGA5P047078 | 0.001928394 | 3.3304128 | down | ENST00000445730 | RP11-525A16.1 |  |
| ASHGA5P043130 | 0.016300811 | 3.3159543 | down | ENST00000435932 | CTB-51J22.1 |  |
| ASHGA5P044675 | 0.006025355 | 3.3119561 | down | ENST00000520156 | RP11-806O11.1 |  |
| ASHGA5P055075 | 0.000306694 | 3.2795173 | down | ENST00000569513 | RP11-660M18.2 |  |
| ASHGA5P039092 | 0.017806564 | 3.2733946 | down | ENST00000469268 | RP11-166N6.2 |  |
| ASHGA5P029148 | 0.011294397 | 3.2476891 | down | ENST00000554196 | LINC00520 |  |
| ASHGA5P022783 | 9.07445E-05 | 3.2457855 | down | ENST00000562678 | RP11-660M18.2 |  |
| ASHGA5P030325 | 2.69542E-05 | 3.2455097 | down | ENST00000560351 | RP11-66B24.4 |  |
| ASHGA5P031038 | 0.002924309 | 3.2049503 | down | ENST00000414816 | AC012317.1 |  |
| ASHGA5P056597 | 0.044871689 | 3.20438 | down | TCONS_00006483 | XLOC_003087 |  |
| ASHGA5P026271 | 0.008034576 | 3.1588107 | down | ENST00000530313 | BDNF-AS |  |
| ASHGA5P018577 | 0.049590508 | 3.1582027 | down | NR_034162 | FLJ43879 |  |
| ASHGA5P026038 | 0.023014938 | 3.1500586 | down | ENST00000530526 | RP11-791J7.2 |  |
| ASHGA5P022727 | 0.000846119 | 3.1191401 | down | ENST00000561596 | RP11-660M18.2 |  |
| ASHGA5P046447 | 0.000977796 | 3.114986 | down | ENST00000420403 | RP11-1L9.1 |  |
| ASHGA5P031839 | 0.01820308 | 3.1132404 | down | ENST00000563811 | RP11-351A20.1 |  |
| ASHGA5P000217 | 1.39413E-06 | 3.112992 | down | chr11:8260275-8275625- | chr11:8260275-8275625 |  |
| ASHGA5P044888 | 0.003212446 | 3.0961828 | down | ENST00000518732 | RP11-706J10.1 |  |
| ASHGA5P041640 | 0.000133929 | 3.0682698 | down | uc003mvq.1 | AX746991 |  |
| ASHGA5P022576 | 0.038177173 | 3.0495639 | down | ENST00000559029 | RP11-279F6.1 |  |
| ASHGA5P042581 | 0.005019695 | 3.0112172 | down | ENST00000425503 | RP1-142L7.5 |  |
| ASHGA5P036474 | 0.000342282 | 2.9779427 | down | ENST00000438633 | AC114765.2 |  |
| ASHGA5P056412 | 3.45552E-05 | 2.9747672 | down | TCONS_00004032 | XLOC_001870 |  |
| ASHGA5P043055 | 0.001784639 | 2.9688712 | down | ENST00000436501 | AC004869.3 |  |
| ASHGA5P032281 | 0.027661059 | 2.9613184 | down | ENST00000577746 | RP11-19P22.8 |  |
| ASHGA5P035955 | 0.03595602 | 2.9569419 | down | ENST00000451622 | AC007099.1 |  |
| ASHGA5P057893 | 0.022950666 | 2.9550099 | down | TCONS_00023350 | XLOC_011212 |  |
| ASHGA5P042476 | 0.001675205 | 2.9432954 | down | ENST00000562834 | RP3-523K23.2 |  |
| ASHGA5P036475 | 0.001111813 | 2.9339125 | down | ENST00000414512 | AC067956.1 |  |
| ASHGA5P019451 | 0.003373101 | 2.9061399 | down | ENST00000485282 | CTB-111H14.1 |  |
| ASHGA5P029166 | 0.003758209 | 2.9056971 | down | ENST00000428508 | RP11-353N4.1 |  |
| ASHGA5P047394 | 0.027655988 | 2.9034412 | down | AI202367 |  |  |
| ASHGA5P047978 | 0.0004369 | 2.8979913 | down | ENST00000533767 | NAV2-AS2 |  |
| ASHGA5P028191 | 0.001287048 | 2.891109 | down | TCONS_00010106 | XLOC_004574 |  |
| ASHGA5P041630 | 0.00420262 | 2.8786249 | down | ENST00000568244 | RP4-668J24.2 |  |
| ASHGA5P051089 | 0.004374139 | 2.8782814 | down | ENST00000465390 | AP000221.2 |  |
| ASHGA5P044385 | 0.001281553 | 2.8612471 | down | ENST00000517884 | RP11-662G23.1 |  |
| ASHGA5P015984 | 0.001927614 | 2.8572679 | down | ENST00000425438 | MLLT4-AS1 |  |
| ASHGA5P030807 | 0.000238187 | 2.8420604 | down | ENST00000431060 | RP11-66B24.2 |  |
| ASHGA5P019084 | 0.030324479 | 2.8311738 | down | ENST00000467484 | RP11-293A21.1 |  |
| ASHGA5P027838 | 0.000615257 | 2.8306175 | down | ENST00000394742 | RP11-392P7.6 |  |
| ASHGA5P016925 | 0.00417816 | 2.8266782 | down | ENST00000435744 | ANKRD30BP2 |  |
| ASHGA5P046902 | 0.006895671 | 2.8265206 | down | ENST00000414295 | RP11-264A11.1 |  |
| ASHGA5P018054 | 0.002949869 | 2.8215747 | down | ENST00000448892 | AC046143.7 |  |
| ASHGA5P034005 | 0.000350272 | 2.8174344 | down | TCONS_00004035 | XLOC_001873 |  |
| ASHGA5P027835 | 0.011399878 | 2.80842 | down | ENST00000540198 | RP11-392P7.6 |  |
| ASHGA5P048903 | 0.018870778 | 2.7979595 | down | TCONS_00024905 | XLOC_011826 |  |
| ASHGA5P032056 | 0.005967979 | 2.7795677 | down | ENST00000574560 | RP11-676J12.8 |  |
| ASHGA5P028764 | 0.001075204 | 2.7590395 | down | ENST00000418076 | RP11-37E23.5 |  |
| ASHGA5P057777 | 0.016479446 | 2.7533594 | down | TCONS_00021948 | XLOC_010545 |  |
| ASHGA5P041901 | 0.010971578 | 2.7388742 | down | NR_026807 | LINC00472 |  |
| ASHGA5P030224 | 0.001113752 | 2.7294362 | down | ENST00000558015 | RP11-561C5.6 |  |
| ASHGA5P058095 | 0.001520808 | 2.7253018 | down | TCONS_00026496 | XLOC_012822 |  |
| ASHGA5P036052 | 0.041414941 | 2.7136339 | down | ENST00000492975 | RP4-752I6.1 |  |
| ASHGA5P033049 | 0.001332487 | 2.7037371 | down | ENST00000583224 | CTC-304I17.5 |  |
| ASHGA5P023421 | 0.00019495 | 2.7017367 | down | ENST00000580612 | RP11-672L10.2 |  |
| ASHGA5P034873 | 0.000341279 | 2.6946414 | down | ENST00000412712 | AC011747.7 |  |
| ASHGA5P019951 | 0.000113371 | 2.6915878 | down | ENST00000505748 | RP11-439E19.3 |  |
| ASHGA5P038952 | 0.001349477 | 2.6912552 | down | TCONS_00024187 | XLOC_011546 |  |
| ASHGA5P026065 | 0.013378468 | 2.6738552 | down | NR_024344 | LOC283174 |  |
| ASHGA5P032342 | 0.001002204 | 2.6737746 | down | ENST00000578040 | RP1-29G21.1 |  |
| ASHGA5P023400 | 0.005625646 | 2.6698262 | down | ENST00000580195 | ANKRD20A5P |  |
| ASHGA5P052068 | 0.002975653 | 2.6669194 | down | ENST00000445097 | FLG-AS1 |  |
| ASHGA5P047922 | 0.005713641 | 2.6594655 | down | ENST00000525154 | RP11-540A21.2 |  |
| ASHGA5P029149 | 0.003496331 | 2.6567851 | down | ENST00000554186 | LINC00520 |  |
| ASHGA5P042433 | 0.006574192 | 2.6408939 | down | NR_047648 | FCGR2C |  |
| ASHGA5P045966 | 0.030565514 | 2.6395348 | down | NR_027406 | LOC100129034 |  |
| ASHGA5P027460 | 3.52514E-05 | 2.6339201 | down | ENST00000551672 | RP11-288D9.1 |  |
| ASHGA5P023401 | 0.004485326 | 2.6287155 | down | ENST00000580995 | ANKRD20A5P |  |
| ASHGA5P030696 | 0.003922526 | 2.6189657 | down | ENST00000559277 | RP11-761I4.3 |  |
| ASHGA5P023293 | 0.004112036 | 2.6166814 | down | ENST00000577614 | ANKRD20A5P |  |
| ASHGA5P036807 | 0.008747948 | 2.6115808 | down | ENST00000433671 | RP5-864K19.4 |  |
| ASHGA5P031012 | 0.003452236 | 2.6094525 | down | ENST00000574654 | CTD-2194A8.2 |  |
| ASHGA5P036456 | 0.036319738 | 2.5989392 | down | uc002vja.1 | AX748340 |  |
| ASHGA5P048291 | 0.002050699 | 2.595285 | down | ENST00000527566 | RP11-167J8.2 |  |
| ASHGA5P034040 | 0.000347172 | 2.5943846 | down | TCONS_00004057 | XLOC_001903 |  |
| ASHGA5P043787 | 0.00936868 | 2.5887768 | down | ENST00000415536 | AC003092.1 |  |
| ASHGA5P045196 | 0.000401064 | 2.5694101 | down | TCONS_00001037 | XLOC_000302 |  |
| ASHGA5P033537 | 0.00805688 | 2.5684821 | down | ENST00000584373 | RP11-627G18.3 |  |
| ASHGA5P045633 | 0.02225642 | 2.5623591 | down | ENST00000450445 | RP11-62F24.2 |  |
| ASHGA5P038245 | 0.037033973 | 2.5616247 | down | NR_045388 | LUST |  |
| ASHGA5P015128 | 0.009465425 | 2.5296341 | down | ENST00000416999 | AC058791.2 |  |
| ASHGA5P044953 | 0.01331624 | 2.5184382 | down | ENST00000499653 | KB-431C1.4 |  |
| ASHGA5P035008 | 0.023615784 | 2.5180879 | down | ENST00000444871 | RP11-89K21.1 |  |
| ASHGA5P035263 | 0.011010647 | 2.5158162 | down | ENST00000584273 | RP11-19E11.1 |  |
| ASHGA5P040939 | 0.048685622 | 2.5011354 | down | uc011cly.2 | BC013821 |  |
| ASHGA5P040408 | 8.98022E-05 | 2.4982399 | down | ENST00000510137 | CTD-2263F21.1 |  |
| ASHGA5P015552 | 0.048383002 | 2.4958058 | down | ENST00000421322 | XIST |  |
| ASHGA5P031709 | 0.027770343 | 2.494638 | down | ENST00000566260 | RP11-989E6.3 |  |
| ASHGA5P035999 | 0.014810133 | 2.487677 | down | uc002suw.1 | BC016831 |  |
| ASHGA5P000014 | 0.010278261 | 2.481877 | down | AF339807 |  |  |
| ASHGA5P027435 | 0.024069812 | 2.4787656 | down | NR_026836 | TRHDE-AS1 |  |
| ASHGA5P035007 | 0.014507071 | 2.4729228 | down | ENST00000430847 | RP11-89K21.1 |  |
| ASHGA5P031135 | 0.002099577 | 2.4715634 | down | ENST00000563019 | RP11-812E19.7 |  |
| ASHGA5P018863 | 0.000956689 | 2.4707151 | down | ENST00000458468 | LINC00478 |  |
| ASHGA5P029013 | 0.004414622 | 2.4617521 | down | ENST00000557232 | AE000662.93 |  |
| ASHGA5P038335 | 0.006045102 | 2.4594045 | down | ENST00000461931 | ST3GAL6-AS1 |  |
| ASHGA5P034905 | 0.005043283 | 2.4569015 | down | ENST00000400892 | NPPA-AS1 |  |
| ASHGA5P000210 | 0.023616143 | 2.4547745 | down | chr11:29287925-29298875+ | chr11:29287925-29298875 | |
| ASHGA5P022524 | 0.008360032 | 2.4525133 | down | NR_038273 | LOC100506874 |  |
| ASHGA5P018727 | 0.003482791 | 2.4452676 | down | ENST00000456937 | BX571672.5 |  |
| ASHGA5P046490 | 0.001004687 | 2.4371975 | down | ENST00000567273 | AL121578.2 |  |
| ASHGA5P040659 | 0.028199881 | 2.433466 | down | ENST00000512185 | CTC-228N24.3 |  |
| ASHGA5P054208 | 0.010046677 | 2.4330429 | down | ENST00000444263 | LINC00278 |  |
| ASHGA5P026708 | 0.007230256 | 2.4322868 | down | ENST00000526206 | CTD-2005H7.1 |  |
| ASHGA5P041087 | 0.016417918 | 2.4189895 | down | uc003jox.1 | AK097288 |  |
| ASHGA5P043112 | 0.049126214 | 2.4165298 | down | ENST00000435148 | RP11-3P22.2 |  |
| ASHGA5P053879 | 0.003816768 | 2.4127574 | down | ENST00000450304 | RP11-611D20.2 |  |
| ASHGA5P044717 | 0.017613845 | 2.4041405 | down | ENST00000448264 | RP11-145A3.1 |  |
| ASHGA5P019699 | 0.017469654 | 2.3950276 | down | NR_033972 | LOC440028 |  |
| ASHGA5P042796 | 0.00032201 | 2.3913861 | down | TCONS_00019306 | XLOC_009139 |  |
| ASHGA5P019809 | 0.000931358 | 2.3910697 | down | ENST00000503483 | CTC-281B15.1 |  |
| ASHGA5P042237 | 0.032664065 | 2.3843892 | down | TCONS_00020826 | XLOC_010114 |  |
| ASHGA5P030225 | 0.005544889 | 2.3754005 | down | ENST00000558365 | RP11-561C5.5 |  |
| ASHGA5P044654 | 0.014899902 | 2.3752421 | down | ENST00000522026 | RP11-177H2.2 |  |
| ASHGA5P022585 | 0.004469608 | 2.3738244 | down | ENST00000559210 | RP11-702M1.1 |  |
| ASHGA5P027242 | 0.001316422 | 2.3734498 | down | TCONS_00011731 | XLOC_005199 |  |
| ASHGA5P047252 | 0.007094714 | 2.3726681 | down | ENST00000420825 | RP1-251M9.2 |  |
| ASHGA5P039397 | 0.027739451 | 2.3684756 | down | NR_049774 | KIAA1324 |  |
| ASHGA5P000065 | 0.02357823 | 2.3679585 | down | AL049275 |  |  |
| ASHGA5P050170 | 0.048722221 | 2.3651283 | down | TCONS_00018656 | XLOC_009007 |  |
| ASHGA5P038151 | 0.004760229 | 2.3622494 | down | ENST00000565519 | RP11-384L8.1 |  |
| ASHGA5P047750 | 0.002313798 | 2.3613136 | down | TCONS_00013627 | XLOC_006291 |  |
| ASHGA5P034102 | 0.002054648 | 2.3590315 | down | ENST00000451054 | RP11-465B22.3 |  |
| ASHGA5P036724 | 0.024086115 | 2.3560478 | down | TCONS_00026523 | XLOC_012847 |  |
| ASHGA5P030805 | 0.005571254 | 2.3538796 | down | ENST00000558254 | RP11-66B24.2 |  |
| ASHGA5P035748 | 0.000142503 | 2.3501062 | down | ENST00000457901 | AC067959.1 |  |
| ASHGA5P051833 | 0.030850457 | 2.34878 | down | NR_047574 | TMEM44-AS1 |  |
| ASHGA5P033773 | 0.043284918 | 2.3486207 | down | ENST00000584414 | RP11-527H14.4 |  |
| ASHGA5P055971 | 0.031840365 | 2.3423976 | down | TCONS_00025721 | XLOC_012542 |  |
| ASHGA5P045942 | 0.027201369 | 2.341149 | down | ENST00000433572 | RP11-542K23.7 |  |
| ASHGA5P047007 | 0.00188462 | 2.340041 | down | ENST00000425267 | RP11-76P2.2 |  |
| ASHGA5P017521 | 0.003385535 | 2.3393316 | down | ENST00000442663 | BX004987.3 |  |
| ASHGA5P018652 | 0.00124479 | 2.3353452 | down | ENST00000456091 | RP11-142G7.2 |  |
| ASHGA5P005138 | 0.002346681 | 2.3349338 | down | uc002iby.2 | LOC388387 |  |
| ASHGA5P016253 | 0.001321856 | 2.3303067 | down | ENST00000428576 | ANKRD20A11P |  |
| ASHGA5P021348 | 0.03556273 | 2.3252067 | down | ENST00000530576 | RP11-266A24.1 |  |
| ASHGA5P034561 | 0.003665767 | 2.3204225 | down | NR_026887 | LOC80054 |  |
| ASHGA5P037418 | 0.009838307 | 2.3194552 | down | ENST00000455275 | AP001439.2 |  |
| ASHGA5P044275 | 0.024017502 | 2.3172529 | down | NR_038236 | LOC100507632 |  |
| ASHGA5P048985 | 0.015261918 | 2.3169778 | down | ENST00000566287 | LA16c-390E6.5 |  |
| ASHGA5P034249 | 0.039096126 | 2.3123354 | down | uc002pda.1 | AX746967 |  |
| ASHGA5P015004 | 0.000368846 | 2.3123188 | down | ENST00000415590 | SFTA1P |  |
| ASHGA5P047773 | 0.001241558 | 2.3117554 | down | ENST00000428273 | RP11-109A6.3 |  |
| ASHGA5P014142 | 0.012030746 | 2.3068115 | down | ENST00000400178 | LINC00478 |  |
| ASHGA5P030921 | 0.014203854 | 2.3005733 | down | ENST00000576810 | RP11-462G12.1 |  |
| ASHGA5P036953 | 0.006031395 | 2.2944256 | down | ENST00000421788 | RP4-697P8.2 |  |
| ASHGA5P041186 | 0.042599744 | 2.292286 | down | ENST00000514114 | CTC-564N23.3 |  |
| ASHGA5P041422 | 0.049389355 | 2.2917256 | down | ENST00000562220 | PCDHA14 |  |
| ASHGA5P027451 | 0.006327679 | 2.2875096 | down | ENST00000547569 | RP11-90C1.1 |  |
| ASHGA5P014664 | 0.003688668 | 2.287058 | down | ENST00000412276 | AC007319.1 |  |
| ASHGA5P027541 | 0.026622097 | 2.2847743 | down | ENST00000549807 | RP11-818F20.5 |  |
| ASHGA5P044029 | 0.033096517 | 2.2847363 | down | NR_027387 | LOC100128822 |  |
| ASHGA5P033310 | 0.012695028 | 2.2819096 | down | ENST00000582940 | RP11-160O5.1 |  |
| ASHGA5P034143 | 0.024912231 | 2.2790228 | down | NR_046201 | LOC400684 |  |
| ASHGA5P043895 | 0.015325502 | 2.2743622 | down | ENST00000562504 | RP11-534L20.5 |  |
| ASHGA5P036997 | 0.001749196 | 2.2743172 | down | ENST00000416638 | RP3-410C9.2 |  |
| ASHGA5P047906 | 0.003139448 | 2.2742213 | down | ENST00000528151 | RP11-236J17.6 |  |
| ASHGA5P042848 | 0.03035146 | 2.2630684 | down | TCONS_00019201 | XLOC_009034 |  |
| ASHGA5P015977 | 0.001551143 | 2.2576984 | down | ENST00000425376 | LINC00113 |  |
| ASHGA5P043365 | 0.004374122 | 2.2561034 | down | ENST00000461145 | RP5-842K16.1 |  |
| ASHGA5P039795 | 0.040208267 | 2.2553765 | down | NR_037863 | LOC285484 |  |
| ASHGA5P025707 | 0.008328309 | 2.2477521 | down | NR_027318 | LOC148145 |  |
| ASHGA5P026324 | 0.008528396 | 2.2399174 | down | TCONS_00014252 | XLOC_006350 |  |
| ASHGA5P039955 | 0.03095973 | 2.2353661 | down | uc003hcu.1 | BC039452 |  |
| ASHGA5P023928 | 0.034546969 | 2.2321448 | down | HMlincRNA971+ | HMlincRNA971 |  |
| ASHGA5P026566 | 0.027108414 | 2.2317127 | down | ENST00000529069 | RP11-119D9.1 |  |
| ASHGA5P026274 | 0.022238066 | 2.2285284 | down | NR_033313 | BDNF-AS |  |
| ASHGA5P031623 | 0.027391588 | 2.2273017 | down | uc021tfl.1 | AF063596 |  |
| ASHGA5P043756 | 0.018976739 | 2.2268307 | down | ENST00000428678 | UPK3BP1 |  |
| ASHGA5P028413 | 0.013432232 | 2.2128376 | down | TCONS_00011088 | XLOC_005087 |  |
| ASHGA5P000016 | 0.000150266 | 2.2084827 | down | AI091628 |  |  |
| ASHGA5P041805 | 0.027372842 | 2.2073052 | down | uc003olu.3 | BC042825 |  |
| ASHGA5P031791 | 0.021442672 | 2.2060093 | down | ENST00000576365 | RP11-212I21.4 |  |
| ASHGA5P055181 | 0.014337749 | 2.2017113 | down | ENST00000453118 | RP1-163G9.1 |  |
| ASHGA5P045373 | 0.024540516 | 2.1982183 | down | ENST00000457720 | RP11-217B7.3 |  |
| ASHGA5P046006 | 0.003264101 | 2.1963155 | down | ENST00000447586 | RP11-409K20.8 |  |
| ASHGA5P040166 | 0.005467275 | 2.1908242 | down | ENST00000504874 | RP11-503L19.1 |  |
| ASHGA5P038627 | 0.020443377 | 2.1894209 | down | ENST00000418353 | RP11-135A1.2 |  |
| ASHGA5P041447 | 0.004092191 | 2.1879264 | down | ENST00000433186 | ARHGAP26-IT1 |  |
| ASHGA5P046251 | 0.023326941 | 2.1854135 | down | uc011mrd.1 | LOC442459 |  |
| ASHGA5P028570 | 0.049754096 | 2.1840791 | down | BE675158 |  |  |
| ASHGA5P015722 | 0.008072331 | 2.1820072 | down | uc010fks.3 | LOC654433 |  |
| ASHGA5P041449 | 0.03020281 | 2.1773535 | down | ENST00000566630 | CTB-85P21.2 |  |
| ASHGA5P033771 | 0.006108054 | 2.1753123 | down | ENST00000578243 | RP11-527H14.4 |  |
| ASHGA5P050440 | 0.004843307 | 2.1743892 | down | NR_037631 | LOC100288911 |  |
| ASHGA5P016935 | 0.016058596 | 2.1720075 | down | ENST00000435867 | RP13-348B13.2 |  |
| ASHGA5P020141 | 0.002493327 | 2.1691548 | down | ENST00000508664 | RP11-164P12.4 |  |
| ASHGA5P019569 | 0.003755082 | 2.1680581 | down | ENST00000492937 | TIPARP-AS1 |  |
| ASHGA5P045579 | 0.003886026 | 2.1673067 | down | TCONS_00001799 | XLOC_001188 |  |
| ASHGA5P028010 | 0.001075715 | 2.1671562 | down | NR_027358 | LINC00592 |  |
| ASHGA5P033022 | 0.037305353 | 2.1632734 | down | ENST00000578883 | RP11-848P1.9 |  |
| ASHGA5P035671 | 0.03364663 | 2.1624089 | down | ENST00000567549 | RP11-341N2.1 |  |
| ASHGA5P047254 | 0.006567592 | 2.1567177 | down | uc001ikr.1 | AF007147 |  |
| ASHGA5P032922 | 0.006886565 | 2.1567083 | down | ENST00000578183 | SNORA59B |  |
| ASHGA5P058395 | 0.000172873 | 2.1563424 | down | uc002nsa.2 | BC068609 |  |
| ASHGA5P029987 | 0.020883143 | 2.1514888 | down | ENST00000560750 | RP11-151N17.2 |  |
| ASHGA5P050594 | 0.030508015 | 2.1474512 | down | ENST00000451400 | AC013402.2 |  |
| ASHGA5P057159 | 0.034117268 | 2.1471659 | down | TCONS_00013857 | XLOC_006488 |  |
| ASHGA5P033528 | 0.009202729 | 2.1410495 | down | ENST00000580867 | RP11-527H14.3 |  |
| ASHGA5P033862 | 0.043381983 | 2.140865 | down | TCONS_00016498 | XLOC_007879 |  |
| ASHGA5P026818 | 0.002168259 | 2.1367914 | down | TCONS_00011820 | XLOC_005303 |  |
| ASHGA5P036826 | 0.015823207 | 2.1366199 | down | uc002xti.1 | AK098067 |  |
| ASHGA5P015206 | 0.018433566 | 2.1340481 | down | NR_015423 | PGM5-AS1 |  |
| ASHGA5P046704 | 0.003891489 | 2.1324675 | down | ENST00000423819 | RP11-297A16.2 |  |
| ASHGA5P033224 | 0.033889262 | 2.129791 | down | NR_038458 | LOC400604 |  |
| ASHGA5P057667 | 0.023609307 | 2.1283131 | down | TCONS_00020551 | XLOC_009864 |  |
| ASHGA5P032106 | 0.007732106 | 2.1276758 | down | ENST00000416958 | AC118754.4 |  |
| ASHGA5P015285 | 0.014052891 | 2.1267695 | down | ENST00000418813 | LINC00478 |  |
| ASHGA5P043962 | 0.023692553 | 2.1213404 | down | TCONS_00018082 | XLOC_008337 |  |
| ASHGA5P026565 | 0.005531454 | 2.1130282 | down | ENST00000511677 | RP11-119D9.1 |  |
| ASHGA5P038675 | 0.011988814 | 2.1118548 | down | ENST00000428171 | AC018832.1 |  |
| ASHGA5P027804 | 0.015727056 | 2.1112904 | down | ENST00000539795 | RP11-90D4.3 |  |
| ASHGA5P047345 | 0.02128323 | 2.1090152 | down | ENST00000446211 | RP11-51B10.2 |  |
| ASHGA5P043821 | 0.004147563 | 2.1057931 | down | TCONS_00018558 | XLOC_008875 |  |
| ASHGA5P019271 | 0.006321588 | 2.1044638 | down | ENST00000475981 | RP11-538P18.2 |  |
| ASHGA5P057280 | 0.002726873 | 2.103947 | down | TCONS_00015224 | XLOC_006719 |  |
| ASHGA5P032192 | 0.014751413 | 2.1028928 | down | ENST00000584017 | RP11-849N15.2 |  |
| ASHGA5P040687 | 0.018199562 | 2.1027524 | down | TCONS_00022099 | XLOC_010709 |  |
| ASHGA5P039363 | 0.031283517 | 2.1024415 | down | NR_037877 | LOC100505912 |  |
| ASHGA5P042240 | 0.02467747 | 2.0979455 | down | ENST00000439891 | RP11-203H2.1 |  |
| ASHGA5P046477 | 0.018488988 | 2.0972256 | down | ENST00000497961 | RP11-242C19.2 |  |
| ASHGA5P057545 | 0.037977711 | 2.0936097 | down | TCONS_00018653 | XLOC_009007 |  |
| ASHGA5P034676 | 0.011538787 | 2.0924914 | down | uc002ped.1 | DKFZp434J0226 |  |
| ASHGA5P035598 | 0.034755423 | 2.0820907 | down | ENST00000396588 | AC097662.2 |  |
| ASHGA5P020319 | 0.00011217 | 2.0814045 | down | ENST00000511495 | CTC-281B15.1 |  |
| ASHGA5P050165 | 0.029160292 | 2.0809884 | down | TCONS_00019067 | XLOC_009007 |  |
| ASHGA5P029765 | 0.000576348 | 2.0803338 | down | ENST00000523671 | MEG3 |  |
| ASHGA5P016234 | 0.018252099 | 2.0802426 | down | ENST00000428329 | AC007319.1 |  |
| ASHGA5P055016 | 0.011696371 | 2.0800662 | down | ENST00000539482 | RP11-807H22.6 |  |
| ASHGA5P038246 | 0.007544899 | 2.0734761 | down | ENST00000421735 | U73167.7 |  |
| ASHGA5P032441 | 0.006657381 | 2.0637855 | down | uc002ibc.3 | BC047651 |  |
| ASHGA5P044635 | 0.015707659 | 2.0616846 | down | ENST00000533615 | FAM66E |  |
| ASHGA5P043426 | 0.022545723 | 2.0602533 | down | uc003wjz.1 | AK055458 |  |
| ASHGA5P039927 | 0.009353441 | 2.0587531 | down | ENST00000441504 | DANCR |  |
| ASHGA5P057555 | 0.000596349 | 2.0587201 | down | TCONS_00018848 | XLOC_008653 |  |
| ASHGA5P028238 | 0.004056967 | 2.0546588 | down | ENST00000552707 | RP11-554E23.4 |  |
| ASHGA5P014800 | 0.016093399 | 2.0524553 | down | ENST00000413472 | RP1-163G9.1 |  |
| ASHGA5P057996 | 0.006691585 | 2.0513652 | down | TCONS_00024756 | XLOC_012040 |  |
| ASHGA5P038334 | 0.01849444 | 2.0509986 | down | NR_046683 | ST3GAL6-AS1 |  |
| ASHGA5P055068 | 0.018177157 | 2.0479752 | down | ENST00000499504 | RP11-736K20.6 |  |
| ASHGA5P036894 | 0.011241233 | 2.0479086 | down | TCONS_00025893 | XLOC_012171 |  |
| ASHGA5P033306 | 0.008695141 | 2.0457636 | down | ENST00000583416 | RP11-583F2.2 |  |
| ASHGA5P044838 | 0.025143313 | 2.0430464 | down | ENST00000523683 | RP11-25K19.1 |  |
| ASHGA5P030923 | 0.013777899 | 2.0386564 | down | ENST00000573220 | RP11-95P2.3 |  |
| ASHGA5P026946 | 0.01994781 | 2.0382351 | down | ENST00000528497 | RP11-555G19.1 |  |
| ASHGA5P034260 | 3.2747E-05 | 2.0342439 | down | uc002pec.3 | BC062328 |  |
| ASHGA5P022660 | 0.008769557 | 2.0342364 | down | ENST00000560461 | RP11-66B24.4 |  |
| ASHGA5P036841 | 0.00634144 | 2.0338313 | down | ENST00000441214 | RP11-290F20.2 |  |
| ASHGA5P056337 | 0.015823776 | 2.0327699 | down | TCONS_00003533 | XLOC_001309 |  |
| ASHGA5P039320 | 0.029984764 | 2.0316558 | down | ENST00000515205 | RP11-539L10.3 |  |
| ASHGA5P055216 | 0.009422786 | 2.0268133 | down | TCONS_00006576 | XLOC_003199 |  |
| ASHGA5P015505 | 0.018851656 | 2.0240122 | down | ENST00000420855 | RP11-561O23.6 |  |
| ASHGA5P039785 | 0.007985612 | 2.0238846 | down | uc003gho.3 | BC042823 |  |
| ASHGA5P045585 | 0.001640997 | 2.0234268 | down | ENST00000442069 | RP11-143M1.2 |  |
| ASHGA5P026688 | 0.017812916 | 2.0232476 | down | ENST00000533528 | RP11-113K21.5 |  |
| ASHGA5P019789 | 0.00015838 | 2.0220761 | down | ENST00000503242 | NREP-AS1 |  |
| ASHGA5P017537 | 0.041328503 | 2.0194116 | down | ENST00000442823 | EPB41L4A-AS1 |  |
| ASHGA5P038926 | 0.013693513 | 2.0171966 | down | uc003dwh.1 | AX747913 |  |
| ASHGA5P029647 | 0.028822862 | 2.0133105 | down | ENST00000555972 | RP3-414A15.2 |  |
| ASHGA5P054291 | 0.012365629 | 2.0132623 | down | TCONS_00013713 | XLOC_006364 |  |
| ASHGA5P053748 | 0.022012424 | 2.0121363 | down | ENST00000438322 | LINC00475 |  |
| ASHGA5P039533 | 0.024063439 | 2.0111622 | down | ENST00000513220 | RP11-710F7.3 |  |
| ASHGA5P047652 | 0.012916334 | 2.0107397 | down | NR_045118 | LOC100289509 |  |
| ASHGA5P047238 | 0.004427389 | 2.0096088 | down | ENST00000561822 | RP11-5N23.3 |  |
| ASHGA5P045785 | 0.0072789 | 2.0061631 | down | ENST00000458111 | RP11-439K3.3 |  |
| ASHGA5P037177 | 0.023651439 | 2.0057257 | down | NR_033967 | SLC2A1-AS1 |  |
| ASHGA5P040164 | 0.008237454 | 2.0045623 | down | ENST00000446530 | BX004987.3 |  |
| ASHGA5P053878 | 0.045457238 | 2.0031245 | down | uc004cib.1 | AX747706 |  |
| ASHGA5P034850 | 0.005886368 | 2.0030717 | down | TCONS_00003566 | XLOC_001341 |  |
| ASHGA5P035771 | 0.032574631 | 2.0019992 | down | ENST00000412749 | AC074117.10 |  |
