# Supplementary material for: Long noncoding RNA ERLR mediates epithelial-mesenchymal transition of retinal pigment epithelial cells and promotes experimental proliferative vitreoretinopathy
Source: Cell Death Differ. 2021 Mar 4;28(8):2351–66. doi: 10.1038/s41418-021-00756-5 (PMC8329214; doi:10.1038/s41418-021-00756-5)
Supplement: Supplementary file 3 — Supplementary File 2 Proteins identification results of IP [file 41418_2021_756_MOESM3_ESM.docx]

**Supplementary File 2**. Proteins identification results of IP sample

| **prot_desc** | **prot_score** | **prot_mass [Da]** | **prot_matches** |
| --- | --- | --- | --- |
| Myosin-9 OS=Homo sapiens GN=MYH9 PE=1 SV=4 | 21291 | 227646 | 757 |
| Vimentin OS=Homo sapiens GN=VIM PE=1 SV=4 | 9229 | 53676 | 399 |
| Isoform 4 of Myosin-10 OS=Homo sapiens GN=MYH10 | 7352 | 233354 | 275 |
| Myosin-10 OS=Homo sapiens GN=MYH10 PE=1 SV=3 | 7332 | 229827 | 274 |
| Actin, cytoplasmic 1 OS=Homo sapiens GN=ACTB PE=1 SV=1 | 7055 | 42052 | 263 |
| Actin, cytoplasmic 2 OS=Homo sapiens GN=ACTG1 PE=1 SV=1 | 6851 | 42108 | 256 |
| Isoform C of Prelamin-A/C OS=Homo sapiens GN=LMNA | 4242 | 65153 | 163 |
| Isoform 8 of Plectin OS=Homo sapiens GN=PLEC | 4120 | 515332 | 194 |
| Prelamin-A/C OS=Homo sapiens GN=LMNA PE=1 SV=1 | 3968 | 74380 | 157 |
| Tubulin beta chain OS=Homo sapiens GN=TUBB PE=1 SV=2 | 3069 | 50095 | 108 |
| Tubulin beta-4B chain OS=Homo sapiens GN=TUBB4B PE=1 SV=1 | 2708 | 50255 | 101 |
| Isoform 2 of Mitochondrial inner membrane protein OS=Homo sapiens GN=IMMT | 2665 | 82973 | 97 |
| POTE ankyrin domain family member E OS=Homo sapiens GN=POTEE PE=1 SV=3 | 2631 | 122882 | 89 |
| Tubulin beta-4A chain OS=Homo sapiens GN=TUBB4A PE=1 SV=2 | 2615 | 50010 | 94 |
| Mitochondrial inner membrane protein OS=Homo sapiens GN=IMMT PE=2 SV=1 | 2460 | 73380 | 90 |
| Voltage-dependent anion-selective channel protein 1 OS=Homo sapiens GN=VDAC1 PE=1 SV=2 | 2296 | 30868 | 80 |
| Unconventional myosin-Ic OS=Homo sapiens GN=MYO1C PE=1 SV=4 | 2289 | 122461 | 97 |
| Isoform 2 of Unconventional myosin-Ic OS=Homo sapiens GN=MYO1C | 2243 | 118632 | 96 |
| Actin, alpha cardiac muscle 1 OS=Homo sapiens GN=ACTC1 PE=1 SV=1 | 2208 | 42334 | 112 |
| Tubulin beta-2A chain OS=Homo sapiens GN=TUBB2A PE=1 SV=1 | 2206 | 50274 | 93 |
| Actin, aortic smooth muscle OS=Homo sapiens GN=ACTA2 PE=1 SV=1 | 2200 | 42381 | 109 |
| Alpha-actinin-4 OS=Homo sapiens GN=ACTN4 PE=1 SV=2 | 2118 | 105245 | 92 |
| DNA-dependent protein kinase catalytic subunit OS=Homo sapiens GN=PRKDC PE=1 SV=3 | 1881 | 473749 | 114 |
| Voltage-dependent anion-selective channel protein 2 OS=Homo sapiens GN=VDAC2 PE=1 SV=2 | 1809 | 32060 | 64 |
| Lamin-B1 OS=Homo sapiens GN=LMNB1 PE=1 SV=2 | 1776 | 66653 | 63 |
