# Supplementary figures and images for: Long noncoding RNA ERLR mediates epithelial-mesenchymal transition of retinal pigment epithelial cells and promotes experimental proliferative vitreoretinopathy
Source: Cell Death Differ. 2021 Mar 4;28(8):2351–66. doi: 10.1038/s41418-021-00756-5 (PMC8329214; doi:10.1038/s41418-021-00756-5)

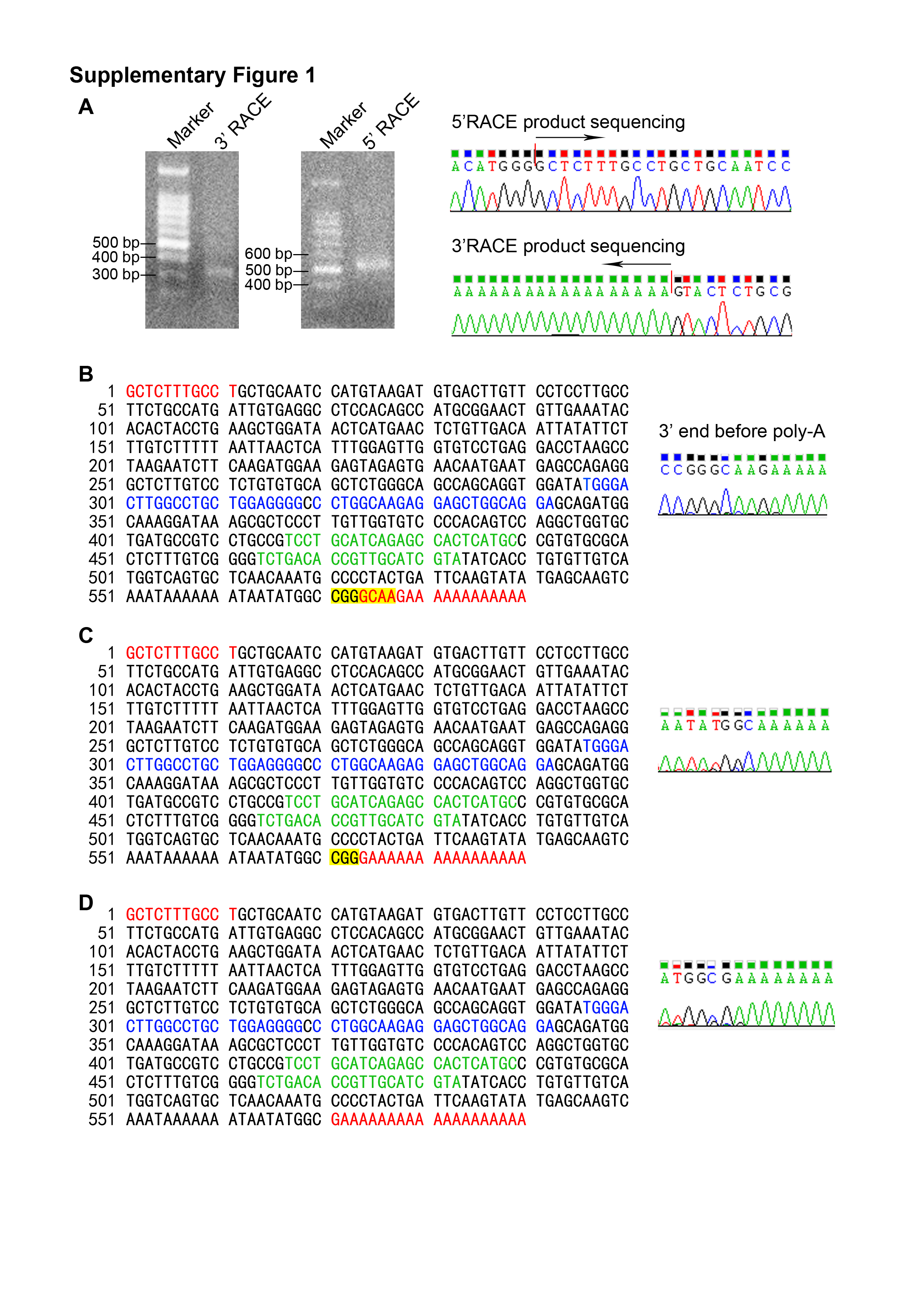

Supplement: Supplementary file 4 — Figure S1 [file 41418_2021_756_MOESM4_ESM.png]

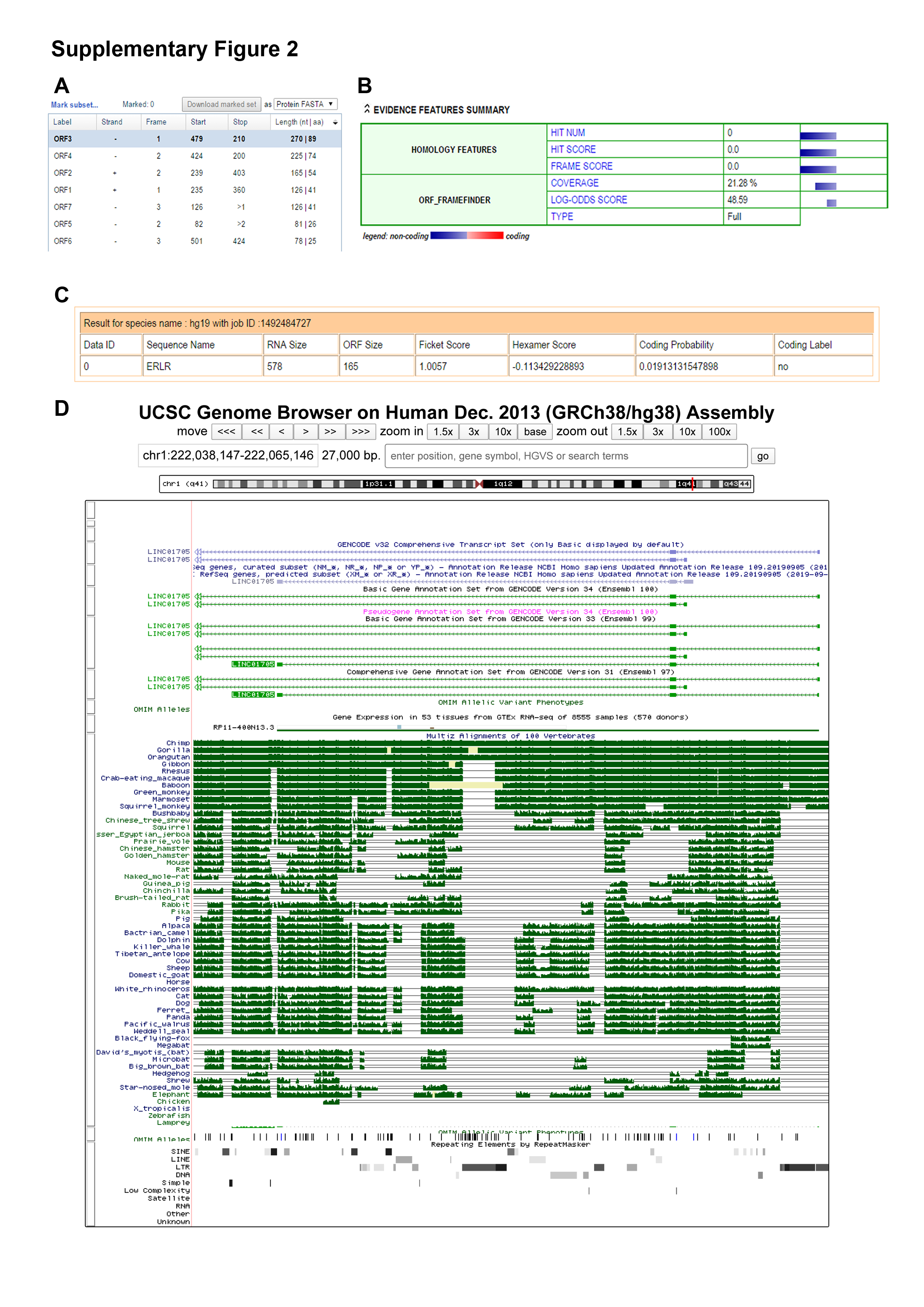

Supplement: Supplementary file 5 — Figure S2 [file 41418_2021_756_MOESM5_ESM.png]

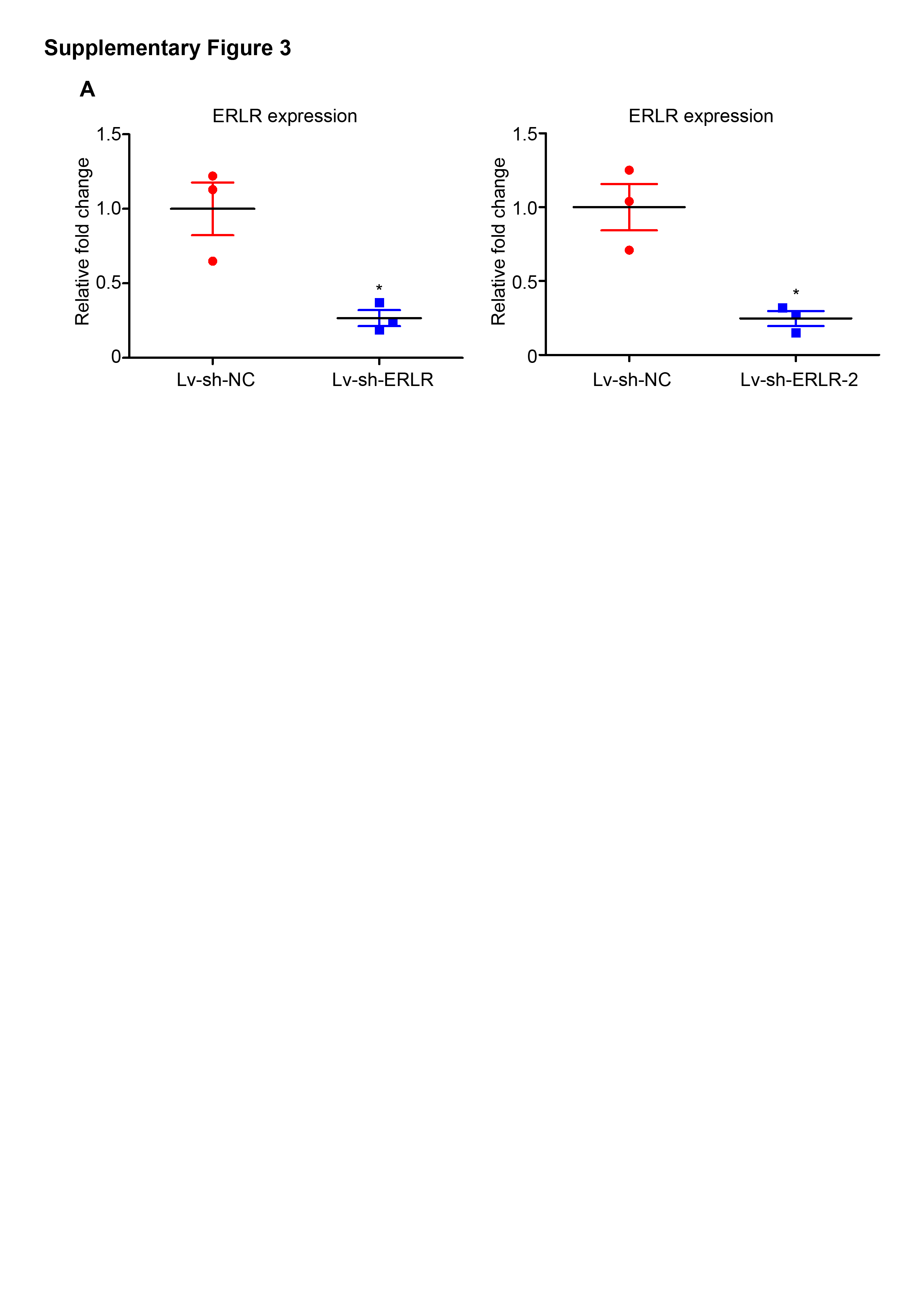

Supplement: Supplementary file 6 — Figure S3 [file 41418_2021_756_MOESM6_ESM.png]

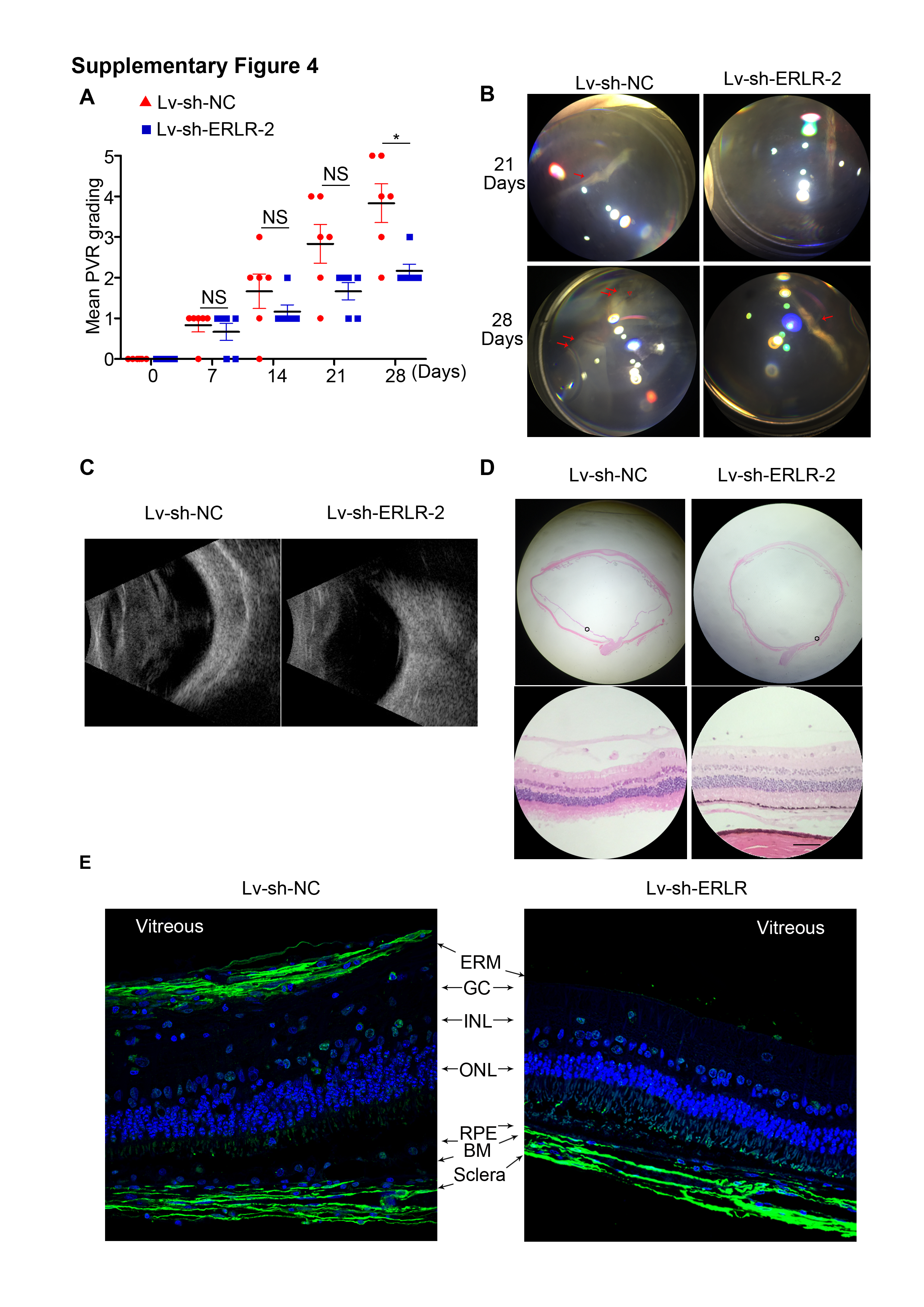

Supplement: Supplementary file 7 — Figure S4 [file 41418_2021_756_MOESM7_ESM.png]

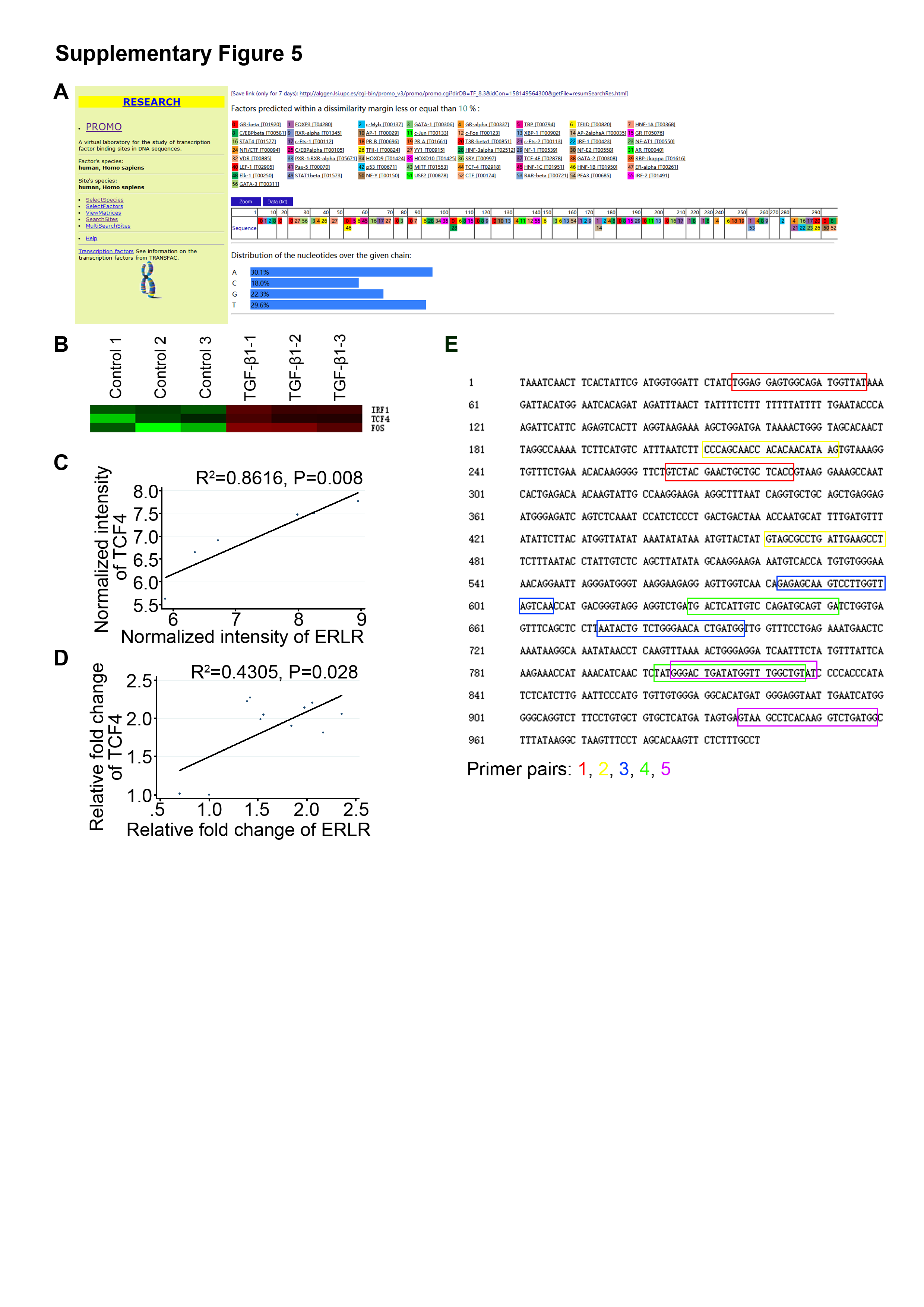

Supplement: Supplementary file 8 — Figure S5 [file 41418_2021_756_MOESM8_ESM.png]

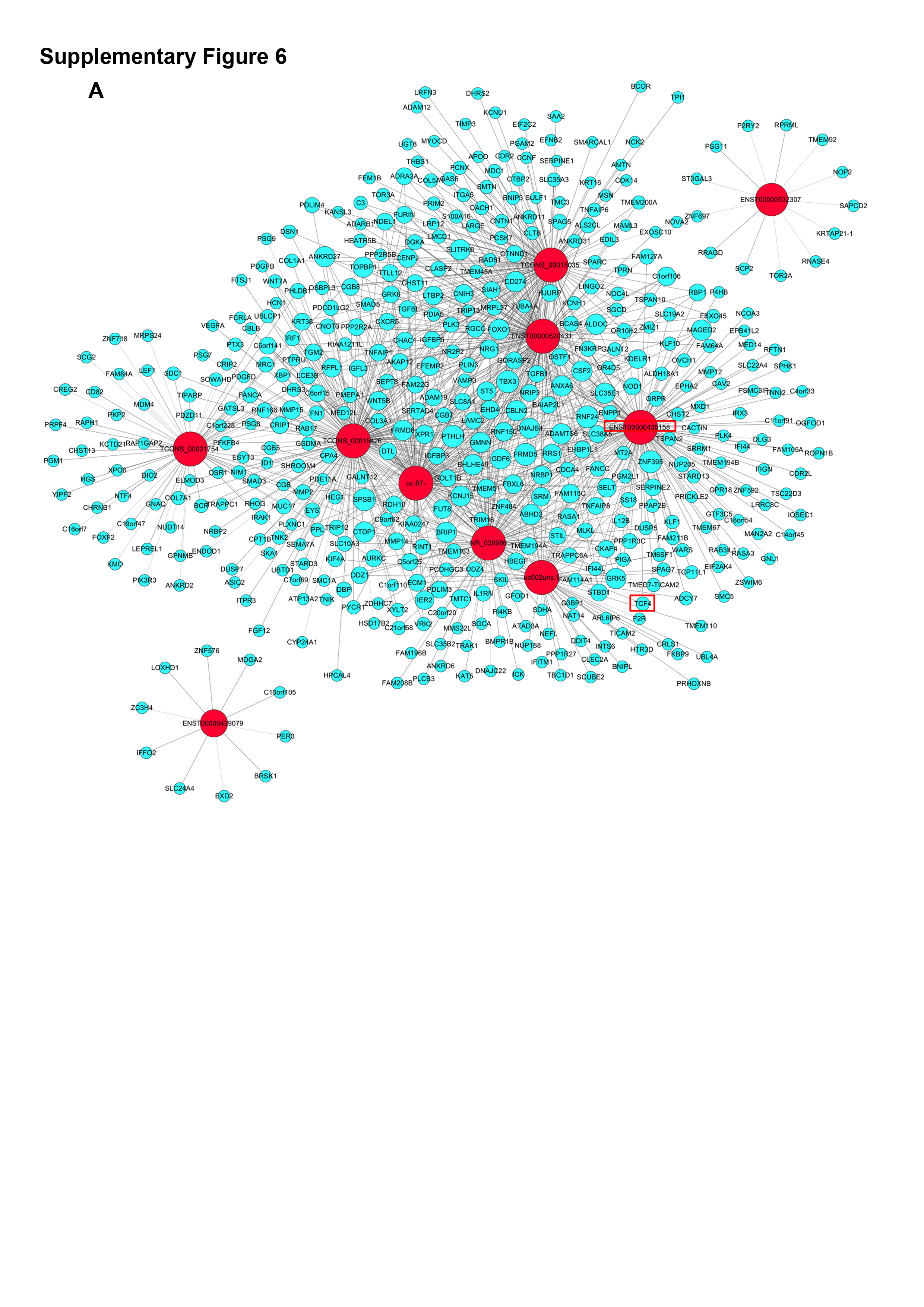

Supplement: Supplementary file 9 — Figure S6 [file 41418_2021_756_MOESM9_ESM.png]

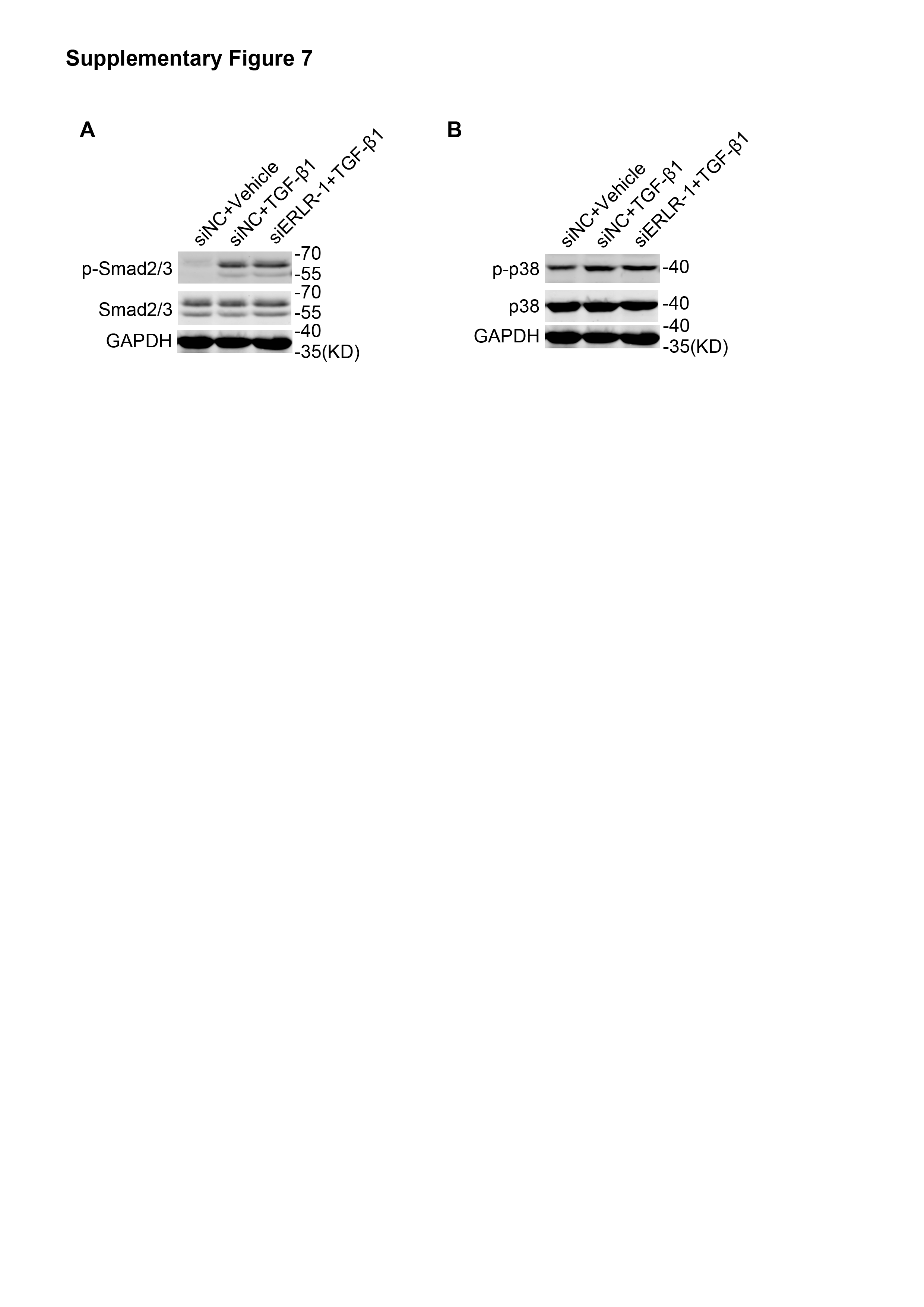

Supplement: Supplementary file 10 — Figure S7 [file 41418_2021_756_MOESM10_ESM.png]
